# Drug Repositioning for Alzheimer’s Disease Based on Systematic ‘omics’ Data Mining

**DOI:** 10.1371/journal.pone.0168812

**Published:** 2016-12-22

**Authors:** Ming Zhang, Gerold Schmitt-Ulms, Christine Sato, Zhengrui Xi, Yalun Zhang, Ye Zhou, Peter St George-Hyslop, Ekaterina Rogaeva

**Affiliations:** 1 Tanz Centre for Research in Neurodegenerative Diseases, University of Toronto, Toronto, Ontario, Canada; 2 Department of Medicine, Division of Neurology, University of Toronto, Toronto, Ontario, Canada; 3 Cambridge Institute for Medical Research, and the Department of Clinical Neurosciences, University of Cambridge, Cambridge, United Kingdom; Istituto di Genetica Molecolare, ITALY

## Abstract

Traditional drug development for Alzheimer’s disease (AD) is costly, time consuming and burdened by a very low success rate. An alternative strategy is drug repositioning, redirecting existing drugs for another disease. The large amount of biological data accumulated to date warrants a comprehensive investigation to better understand AD pathogenesis and facilitate the process of anti-AD drug repositioning. Hence, we generated a list of anti-AD protein targets by analyzing the most recent publically available ‘omics’ data, including genomics, epigenomics, proteomics and metabolomics data. The information related to AD pathogenesis was obtained from the OMIM and PubMed databases. Drug-target data was extracted from the DrugBank and Therapeutic Target Database. We generated a list of 524 AD-related proteins, 18 of which are targets for 75 existing drugs—novel candidates for repurposing as anti-AD treatments. We developed a ranking algorithm to prioritize the anti-AD targets, which revealed CD33 and MIF as the strongest candidates with seven existing drugs. We also found 7 drugs inhibiting a known anti-AD target (acetylcholinesterase) that may be repurposed for treating the cognitive symptoms of AD. The CAD protein and 8 proteins implicated by two ‘omics’ approaches (ABCA7, APOE, BIN1, PICALM, CELF1, INPP5D, SPON1, and SOD3) might also be promising targets for anti-AD drug development. Our systematic ‘omics’ mining suggested drugs with novel anti-AD indications, including drugs modulating the immune system or reducing neuroinflammation that are particularly promising for AD intervention. Furthermore, the list of 524 AD-related proteins could be useful not only as potential anti-AD targets but also considered for AD biomarker development.

## Introduction

Alzheimer’s disease (AD) is the most common form of dementia (6% of people above age 65 [1]), affecting ~48 million people worldwide in 2015 according to the world health organization. AD brain pathology is characterized by neuronal tau inclusions and amyloid plaques, mainly consisting of Aβ_40/42_ peptides generated by the cleavage of APP protein. Aβ_42_ peptide is occurring in a tenth of the amount of Aβ_40_, but aggregates faster than Aβ_40_ and is more toxic in cell culture assays [2]. The Aβ accumulation is an early event that could trigger downstream events (e.g., misprocessing of the tau protein and brain inflammation) [3]. AD is one of the most costly chronic diseases, with a global cost of $605 billion as estimated by the World Alzheimer's Association. So far, there are 5 FDA approved drugs on the market according to the Alzheimer’s Association, but none of them can cure AD. There is an urgent need to develop novel anti-AD therapies, however traditional drug development takes a long time (10–17 years), requires massive financial investments, and yet is burdened by a very low success rate (~0.4% for AD from year 2001 to 2012 [4, 5]). Drug repositioning (repurposing) is used to redirect approved and clinical trial drugs for treating another disease [6]. It is an attractive strategy to pursue for AD [7] that can dramatically reduce drug development time, cost and safety risk, because drug toxicity data are often available from former phase I/II clinical trials.

Previous studies have applied various methods of analyzing ‘omics’ data to identify promising drugs for repurposing, including comparison analyses of gene expression patterns (connectivity maps) [8], text mining [9], network analyses [10], exploration of data from genome wide association studies (GWASs) [11] and the analysis of pathogenesis knowledge from the Online Mendelian Inheritance in Man (OMIM) database [12]. In addition, computational methods have been used to predict drug-protein interactions [13], drug off-targets [14], drug side effects [15] and drug-disease associations [16]. Our group recently developed a comprehensive drug repositioning strategy based on mining genomic, proteomic and metabolomic data that revealed 9 drugs with new anti-diabetes indications [6]. In the current study, we used an improved approach that added epigenomic data and a ranking strategy for anti-AD drug repositioning.

Most AD patients have sporadic late-onset disease, and are free from rare mutations in known causal AD genes (*APP*, *PSEN1* and *PSEN2*) [3]. Sporadic AD is associated with multiple genetic variations of small effect (e.g., most GWAS loci) or moderate effect (e.g., *APOE*-ε4 [17] and *TREM2* rs75932628 T-allele [18, 19]), and could be influenced by other risk factors (e.g., head trauma [20], diabetes [21] and aging [22]). The complex interactions between genetic and environmental factors lead to alterations in proteins, metabolites and epigenetic modifications in the brain tissue and/or body fluids of AD patients.

The large amount of biological data accumulated to date warrants comprehensive investigation to better understand AD pathogenesis and facilitate the process of anti-AD drug repositioning. Hence, the current study aimed to systematically analyze AD-related ‘omics’ data to discover potential anti-AD drug targets, develop an algorithm to rank these drug targets, and suggest a priority for repurposing existing drugs as potential anti-AD therapies.

## Materials and Methods

### Database search for potential anti-AD targets

We searched the NHGRI-EBI GWAS Catalog (http://www.ebi.ac.uk/gwas) to extract AD-associated genetic variations; and the Human Metabolome Database (HMDB) to extract AD-related metabolites. To shortlist AD-related proteins and epigenetic changes, we searched the PubMed database up to June 2016 using the keywords: “Alzheimer’s disease and proteomics”, “Alzheimer’s disease and protein/proteomics”, “Alzheimer’s disease and DNA methylation”, “Alzheimer’s disease and epigenetics”. We incorporated this literature in our study according to the following criteria: 1) all samples (e.g., serum, plasma, urine or tissue) had to be human; 2) the disease diagnosis had to be “Alzheimer’s disease” or “Late-onset Alzheimer’s disease”; and 3) for proteins, all samples had to be CSF.

For the GWASs, we extracted information on 1) genes; 2) SNPs; 3) initial sample size; 4) replication sample size; 5) p-value; 6) effect size: odds ratio (OR) or beta-coefficients; 7) PubMed ID. For the epigenetic studies, we extracted information on 1) protein ID; 2) gene ID; 3) patient status; 4) sample size; 5) platform; 6) PubMed ID. For the proteomics studies, we extracted information on 1) protein name; 2) gene name; 3) Uniprot ID; 4) sample type; 5) patient status; 6) sample size; 7) platform; 8) PubMed ID. For the metabolomics studies, we extracted information on 1) metabolite; 2) sample type; 3) concentration in patients; 4) patient status; 5) age; 6) gender; 7) PubMed ID.

### Mapping AD-related metabolites to proteins and visualizing the metabolite-protein network

We extracted the names of proteins that linked to AD-related metabolites based on the HMDB database. To visualize the association between these metabolites and the proteins affecting them, we constructed a metabolite-protein network using Cytoscape software v3.3.0 (www.cytoscape.org) [23].

### Mapping AD-related proteins to existing drugs

We selected a panel of AD-related proteins retrieved from GWASs, epigenetic and proteomics studies, as well as proteins linking to ≥2 AD-related metabolites retrieved from the HMDB. To establish a link between these AD-related proteins to drugs, we used two public databases: the Therapeutic Target Database (TTD version 4.3.02) containing information on the 236 targets of 20,667 approved, clinical trial and experimental drugs [24], and the DrugBank database (www.drugbank.ca) containing 4,800 drug entries including >1,350 FDA-approved small molecule drugs, 123 FDA-approved biotech (protein/peptide) drugs, 71 nutraceuticals and >3,243 experimental drugs [25]. To focus on the most promising drugs that might be repurposed for treating AD, only target-drug pairs comprising drugs that were either approved or had been examined in clinical trials were selected. From these two drug databases, we extracted information on 1) drug target name; 2) drug name; 3) original drug indication; 4) drug stage; and 5) drugs’ modes of action.

### Information on pathogenesis and the drugs’ modes of action for anti-AD drug repositioning

We extracted knowledge about pathogenesis of potential anti-AD targets from the OMIM database (http://www.omim.org) and a PubMed literature search. We obtained information on the gain of function (GOF) or loss of function (LOF) roles of the drug targets in humans or animal models. Target pathogenesis information together with the drugs’ modes of action retrieved from the drug databases were used to rationally shortlist promising anti-AD drugs.

### The ranking algorithm of anti-AD drug targets

To prioritize potential anti-AD drug targets, we developed an algorithm to score the targets. To calculate the target score, a weighted sum model [26] was used that employed three criteria: 1) the level of change of the AD-related proteins that were presented by fold changes of proteins or the OR of minor alleles; 2) the number of citations of the paper that reported the AD pathogenesis of the target based on Google scholar; 3) the number of publications that reported the target in connection to AD based on the PubMed search. To more comprehensively consider both the confidence of disease-target association (criteria 1 and 3) and the strength of evidence in support for AD pathogenesis (criteria 2); we gave each criterion equal weighted values. For the targets retrieved from metabolomics, we estimated the fold change of the target based on the fold changes of the corresponding metabolites adjusted to the total number of metabolites connected to that target, assuming other linked metabolites did not change. We also used internal controls to adjust the target scores to known AD-related proteins/genes: 1) the fold changes of proteins were adjusted to the fold change (2.37) of Aβ_42_ in CSF of AD patients [27]; 2) the OR of risk alleles were adjusted to the OR (3.7) of *APOE*-ε4 allele vs. ε3 allele (ALZforum); 3) the number of citations was adjusted to the number of times the first paper reporting the segregation of an *APP* mutation with familial AD [28] was cited (4092, up to Feb 2016); 4) the number of publications was adjusted to the number of articles with both APP and AD as keywords (11294, up to Feb 2016). We used the equations (Eqs 1, 2 and 3) to estimate the target scores of those targets retrieved from metabolomics (TSm), proteomics (TSp) and genetics (TSg).

$$\text{TSm}=0.33\times \frac{\sum_{\text{i}=1}^{\text{n}}\left|\text{Fi}\right|+\text{N}-\text{n}}{\text{N}\times 2.37}+\frac{0.34\times \text{C}}{4092}+\frac{0.33\times \text{H}}{11294};$$ (1)

$$\text{TSp}=0.33\times \frac{\left|\text{F}\right|}{2.37}+\frac{0.34\times \text{C}}{4092}+\frac{0.33\times \text{H}}{11294};$$ (2)

$$\text{TSg}=0.33\times \frac{\text{OR}}{3.7}+\frac{0.34\times \text{C}}{4092}+\frac{0.33\times \text{H}}{11294};$$ (3)

where F = fold change of the proteins or metabolites between AD and normal controls (positive if the AD group is higher than the control group, negative if the AD group is lower than the control group); N = total number of proteins connected to the metabolite, n = number of proteins connected to the AD-related metabolite; C = the number of citations of the target pathogenesis paper; H = the number of publications reporting both AD and the target; OR = the odds ratio of the risk allele.

### Bioinformatics analyses

Protein-protein interactions of AD-related proteins were analyzed using the String tool (http://string-db.org) by selecting “experiments” as active prediction method. Cytoscape software v3.3.0 was used to visualize the protein-protein interaction network. The pathway enrichment analysis was conducted using the David online tool (https://david.ncifcrf.gov/) by selecting the KEGG database. Benjamini corrected p-values <0.05 were considered significant.

### Computational analysis of candidate drug targets and repurposed drugs

To validate the anti-AD drug targets derived from our ‘omics’ mining method, we used the Toppgene tool (https://toppgene.cchmc.org), which ranks candidate genes based on functional similarity to the training genes, and the Toppnet tool (https://toppgene.cchmc.org), which ranks candidate genes based on topological features in protein-protein interaction networks and their similarity to the training genes [29]. In the current study, we used 5 training genes selected based on the strongest AD risk-effect (*APP*, *PSEN1*, *PSEN2*, *APOE*, and *TREM2*).

We also used two online resources (Connectivity Map (Cmap), http://portals.broadinstitute.org/cmap/; and C2Maps, http://rdc02.uits.iu.edu:7777/pls/apex/f?p=208:1:2695462252197431::NO) to analyze the small molecule drugs of the repurposed drugs. Using Cmap, we analyzed whether the change in the pattern of gene expression is similar between the repurposed drugs and known anti-AD drugs (memantine and galantamine) [6]. While C2maps assessed the anti-AD drug and gene association; based on network mining, literature mining, and drug effect annotation [30].

## Results

### Systematic mining of ‘omics’ data revealed potential AD-related proteins

**Fig 1:**
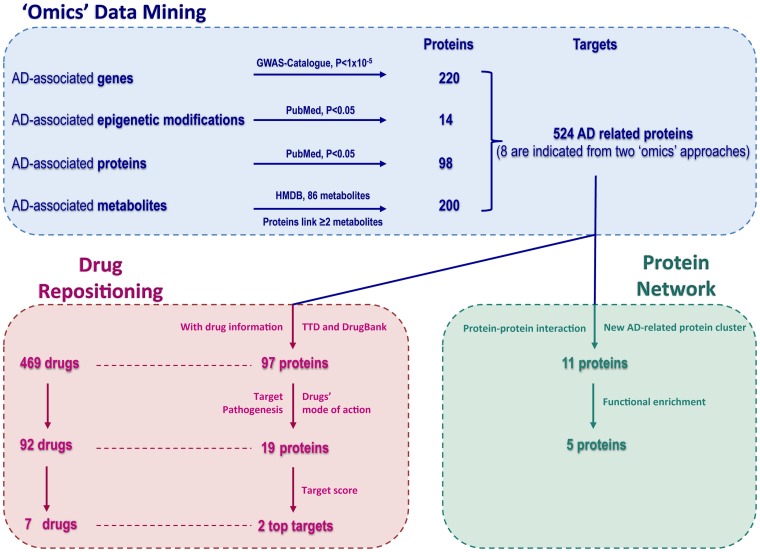
Flow-chart of the drug repositioning strategy for AD based on ‘omics’ data mining. We searched the GWAS Catalogue, PubMed, and HMDB database, and extracted 244 genetic variations, 14 epigenetic modifications, 98 proteins and 86 metabolites associated with AD. We also extracted 1179 protein-metabolite interactions based on the HMDB database and found 200 proteins linked to ≥2 AD associated metabolites. In total, we shortlisted 524 AD-related proteins, 8 of which were revealed by 2 ‘omics’ approaches. By using the TTD and DrugBank database, we extracted information on drugs, targets and the drugs’ mode of action. Considering AD pathogenesis together with the drugs’ mode of action, we found 19 targets of 92 drugs with anti-AD indication that may be repurposed. We then scored these targets and found CD33 and MIF to be the two highest ranked targets. A protein-protein interaction analysis of 524 AD-related proteins detected a novel network of 11 proteins with CAD as a hub protein (functional enrichment analysis revealed that 5 of these 11 proteins are involved in the “Alanine, Aspartate and Glutamate Metabolism” pathway presented in Fig 3).

**Fig 2:**
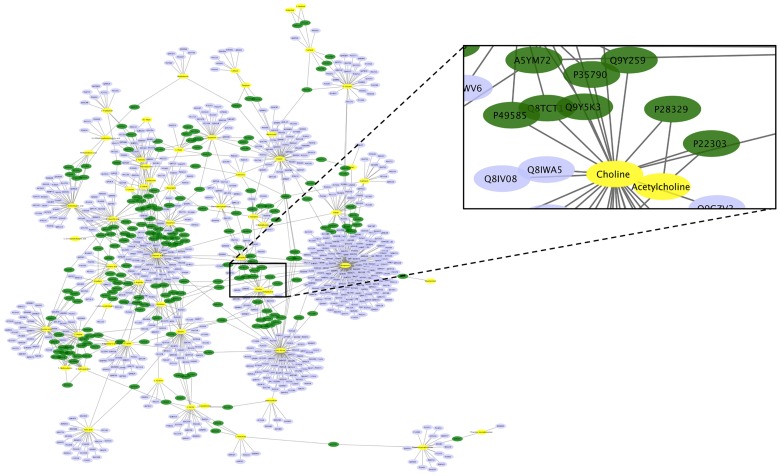
AD related protein-metabolite network. 1179 protein-metabolite interactions were indicated from the HMDB database. The zoomed-in inset shows that acetylcholinesterase (P22303), a known anti-AD target, interacts with 2 AD-related metabolites (Choline and Acetylcholine). The nodes with yellow color represent metabolites that were altered in AD patients, the nodes with purple color represent proteins that linked to AD associated metabolites, and the nodes with green color represent proteins that linked to ≥2 AD associated metabolites.

We analyzed 4 epigenetic, 7 proteomic and 18 metabolomic studies, as well as 31 GWASs; and retrieved 14 epigenetic events associated with AD, as well as 98 proteins and 86 metabolites that were reported to be significantly altered in AD patients, and 244 genetic variations associated with AD implicating 220 genes (Fig 1, S1–S4 Tables). Based on the HMDB, 200 proteins were linked to ≥2 AD-related metabolites (1179 metabolite-protein pairs). The AD-related metabolite-protein network (Fig 2) shows highly interconnected metabolic pathways of various metabolites.

In total, ‘omics’ data revealed 524 unique AD-related proteins, including 8 proteins that showed alterations in two platforms (S5 Table). Among them, ABCA7, APOE, BIN1 and PICALM had reports on AD-related functional studies, while findings related to CELF1, INPP5D, SPON1 and SOD3 encourage further analysis regarding their roles in AD pathogenesis.

**Fig 3:**
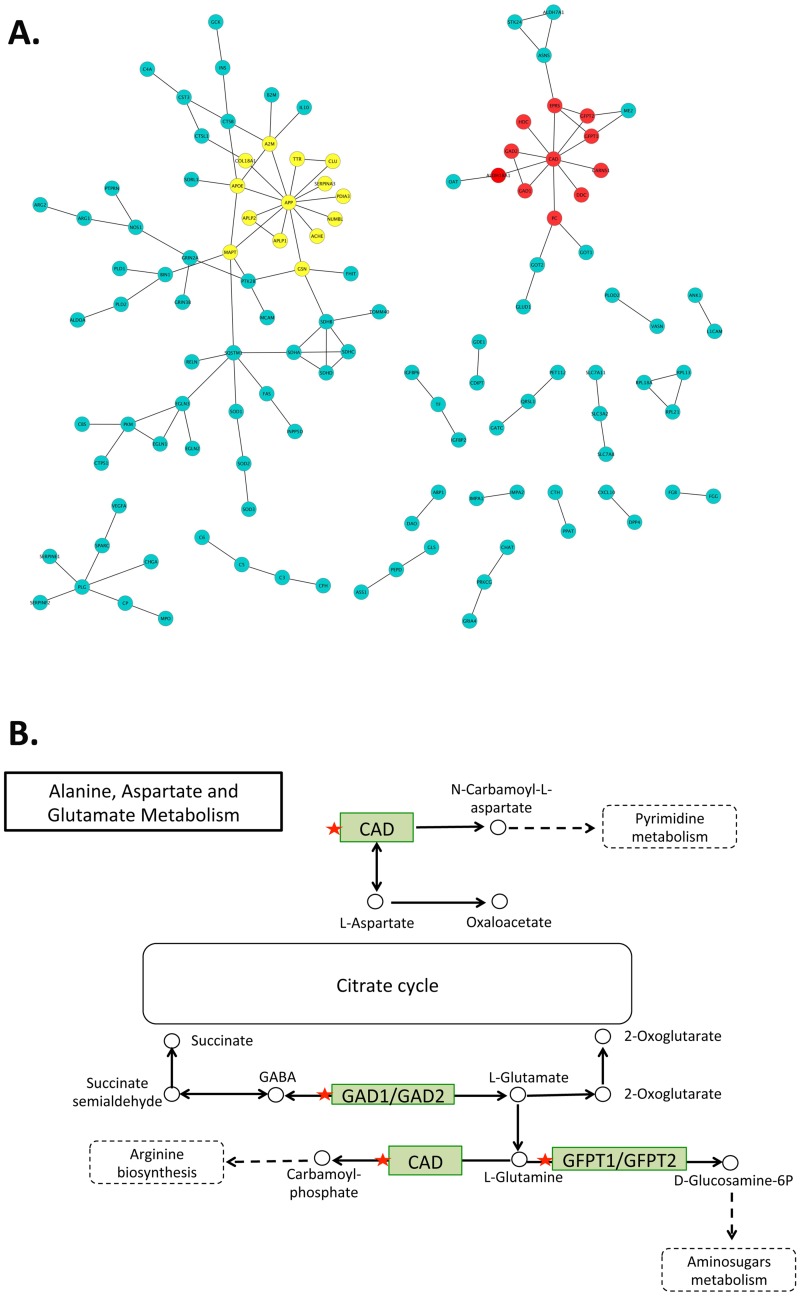
**A)** Protein-protein interaction analysis of 524 AD-related proteins revealed two large protein clusters: the APP network (14 yellow nodes) and the CAD network (11 red nodes). **B)** The functional enrichment analysis found that five proteins (labeled with red stars) of the CAD network are involved in the “Alanine, Aspartate and Glutamate Metabolism” pathway (CAD, GAD1, GAD2, GFPT1, GFPT2). The figure was generated based on the results obtained by the David online tool and the KEGG database.

The protein-protein interaction analysis of 524 AD-related proteins detected two core hub proteins: APP (encoded by causal AD gene) and CAD (Fig 3A). CAD links to another 10 proteins, all of which are associated with AD-related metabolites. The pathway enrichment analysis revealed that these 11 proteins are significantly enriched in the “Alanine, Aspartate, Glutamate metabolism” pathway (Benjamini corrected p-value = 0.000002) (Fig 3B), with 5 proteins (GAD1, GAD2, GFPT1, GFPT2 and CAD) involved in this pathway.

### Drugs with possible anti-AD indication based on knowledge of drugs’ modes of action and AD pathogenesis

Searching the TTD and DrugBank databases using Uniprot IDs for the aforementioned 524 protein targets revealed that 19 of them (with information on AD pathogenesis) were linked to 92 approved or clinical trial drugs (with data on drugs’ modes of action), supporting their potential anti-AD roles, such as reducing cognitive impairment or increasing neuron protection and Aβ clearance [Table 1, S6 Table]. Two of these 19 proteins, acetylcholinesterase (ACHE) and APP, are known anti-AD drugs targets, corresponding to 17 existing drugs [Table 1, S6 Table], including three approved drugs for AD treatment (galantamine, rivastigmine and donepezil). This validates the ability of our strategy to detect known anti-AD drugs and supports its potential to discover novel anti-AD indications of existing drugs. Apart from APP, we found 18 potential anti-AD targets with 75 existing drugs that might have a novel anti-AD indication [S6 Table]. Of note, 7 drugs targeting acetylcholinesterase were not previously used for treating AD symptoms and could be repurposed for anti-AD therapy.

**Table 1:** 'Omics' data mining revealed potential anti-AD drug targets from existing approved and clinical trial drugs.

Uniprot ID	Database	Target name	Target score	Target source	Number of drugs
P20138	TTD	Myeloid cell surface antigen CD33	0.715	GWAS	6
P14174	TTD	Macrophage migration inhibitory factor	0.438	Proteomics	1
P22303	TTD/DrugBank	Acetylcholinesterase*	0.384	Metabolomics	10
Q96KS0	TTD	Hypoxia-inducible factor-prolyl hydroxylase	0.319	Metabolomics	4
O43497	TTD	Voltage-dependent T-type calcium channel alpha-1G subunit	0.291	GWAS	6
P00747	TTD/DrugBank	Plasminogen	0.192	Proteomics	7
P21728	TTD/DrugBank	Dopamine D1 receptor	0.171	Metabolomics	13
P00325	DrugBank	Alcohol dehydrogenase 1B	0.159	Metabolomics	1
P01009	TTD	Alpha-1-antitrypsin	0.155	Proteomics	3
P35228	TTD	Nitric oxide synthase, inducible	0.146	Metabolomics	3
P05164	TTD	Myeloperoxidase	0.144	Metabolomics	2
P15692	TTD	Vascular endothelial growth factor A	0.144	Proteomics	2
P15121	TTD/DrugBank	Aldose reductase	0.139	Metabolomics	6
O76074	TTD	CGMP-specific 3',5'-cyclic phosphodiesterase	0.138	Metabolomics	6
O14939	DrugBank	Phospholipase D2	0.137	Metabolomics	1
P21917	DrugBank	Dopamine D4 receptor	0.133	Metabolomics	3
P21964	TTD/DrugBank	Catechol-O-methyl-transferase	0.13	Metabolomics	3
P10635	TTD	Cytochrome P450 2D6	0.128	Metabolomics	1
P05067	TTD	Amyloid precursor protein*	1.000	Proteomics	14

* Represents known anti-AD target

### The ranking algorithm revealed two promising anti-AD drug targets

**Fig 4:**
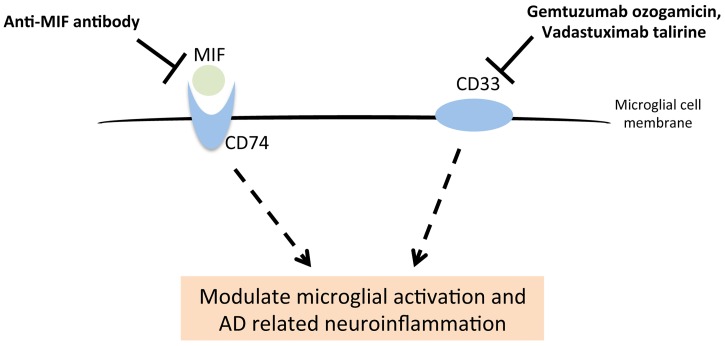
The top two anti-AD targets, MIF and CD33, affect microglial activation. Both CD33 and the MIF receptor (CD74) are expressed on the microglial cell surface. Antibodies/inhibitors of MIF and CD33 may be assessed for their effects in modulating AD-related neuroinflammation.

We developed a ranking algorithm to prioritize the anti-AD targets (APP was set as an internal control with a target score of 1); and determine which of the drugs targeting these proteins are the most promising to pursue in validation studies. We evaluated our algorithm using three known anti-AD drug targets, acetylcholinesterase, TREM2 and APOE, which revealed a medium/high target score of 0.384, 0.459 and 0.887, respectively [S7 Table]. The mean target score for the 17 novel anti-AD targets is 0.235, ranging from 0.143 to 0.782 [S7 Table]. There are two targets with scores greater than that of acetylcholinesterase: CD33 (0.782) and MIF (0.438), both of which are linked to microglial activation and neuroinflammation [Fig 4]. Antibodies/inhibitors targeting CD33 and MIF were originally tested in clinical trials for the treatment of acute myelogenous leukemia (AML) or solid tumors [S6 Table]. Our results suggest that they might also be good candidates for treating AD-related neuroinflammation [Fig 4].

Another two targets (HIF and CACNA1G) had medium target scores of 0.345 and 0.319. Ten small molecule drugs targeting these two proteins might also be repurposed for treating AD [S6 Table], and warrant further validation.

### Computational analysis validated top ranked anti-AD drug targets

Medium/high Toppgene scores (>0.6) and Toppnet scores (>1E-05) were observed for 8 of the top 10 anti-AD drug targets (S7 Table), suggesting that our ranking algorithm corresponds well to other ranking methods. Toppgene scores for the top 3 targets are 0.67 (CD33), 0.71 (MIF) and 0.83 (ACHE). The Toppnet scores for the top 3 targets are 6.7E-05 (CD33), 1.4E-05 (MIF) and 2.8E-05 (ACHE).

The evaluation of our top candidate drugs (antibodies targeting CD33 and MIF) is important but lacks the appropriate computational methods to rank antibody drugs. Here, we used two online resources (Cmap and C2maps) to analyze the small molecule drugs of the repurposed drugs. Using Cmap, we found that only one drug (edrophonium) showed positive correlation to memantine (enrichment score = 0.62, p = 0.05). Other drugs had non-significant results or no gene expression information in Cmap. Using C2maps, we found that only one drug (physostigmine) had a high protein ranking score of 0.99 and low enriched drug p-value (p = 2E-23). C2maps also validated 3 known anti-AD drugs, including galantamine (protein ranking score = 0.99, enriched drug p = 4.7E-4), rivastigmine (protein ranking score = 0.99, enriched drug p = 1.2E-10) and donepezil (protein ranking score = 0.99, enriched drug p = 5.8E-9). The scores of other drugs are not available in C2maps.

## Discussion

In the current study, we improved our ‘omics’-based drug repositioning strategy [6] by adding epigenetic data into the search for drugs to be repositioned for AD. Epigenetic modifications, especially DNA methylation, have been reported to be associated with aging [31], [32], AD [33], and Parkinson’s disease [34]. Furthermore, genetic variations that modulate DNA methylation age may control biological aging [35], which is the strongest risk factor for AD. Overall, we revealed 524 AD-related proteins, 18 of which are targets for 75 existing drugs making them novel candidates for repurposing as anti-AD treatments. Importantly, 8 AD-related proteins were implicated by two ‘omics’ approaches, suggesting their priority as anti-AD targets [S5 Table]. Of note, 4 of them (CELF1, INPP5D, SPON1 and SOD3) do not have information on AD pathogenesis and need to be further investigated in functional studies.

The list of 524 AD-related proteins could be useful not only as potential anti-AD targets, but also considered for developing AD biomarkers. Moreover, the protein-protein interactions among these 524 proteins point to a core hub CAD protein connected to 10 proteins, each of which is associated with ≥2 AD-related metabolites. A pathway analysis of the CAD hub suggested an enriched “Alanine, Aspartate, Glutamate metabolism” pathway. Further investigations may be initiated to design drugs targeting CAD to modulate the AD-related imbalance in neurotransmitters (e.g., glutamate [36] or GABA [37]). The comprehensive analyses of multiple “omics” data provide a unique opportunity to understand the most-relevant biomarkers/risk factors related to AD, thereby facilitating the process of identifying protein targets and drugs for repurposing.

We also improved our ‘omics’-based drug repositioning strategy by developing a ranking algorithm to prioritize the drug targets. Previous scoring algorithms, such as calculating the confidence of drug-protein interactions [38] and disease–disease, drug–drug and target–target relationships (constructed based on their similarities) [16], evaluated the strength of the association but not the therapeutic rationale based on pathogenesis information of the target and the action mode of the drug. The current study employed a ranking algorithm that considered both the strength of the target-disease association and the quality of the study related to AD pathogenesis of a particular protein (based on number of citations), therefore providing a target score considering therapeutic rationale. To validate our ranking method, we used the online tools, Toppgene and Toppnet, to analyze the targets’ functional and topological similarity to known AD genes. The results of our ranking algorithm are reliable, because 8 of our top 10 targets had medium/high scores from Toppgene and Toppnet.

Using a computational method to evaluate our top repurposed drugs (antibodies targeting CD33 and MIF) is difficult, because most available computational tools are used for small molecule drugs. Cmap and C2maps revealed two drugs of interest: edrophonium and physostigmine, both of which are ACHE inhibitors. Other repurposed small molecule drugs cannot be properly evaluated using Cmap and C2maps, because these two methods used the only known anti-AD target (ACHE) to assess similarity, and thereby may have limitations when evaluating other targets that have quite different pathogenic mechanisms. In the future, experimental validation is needed to evaluate the efficacy and toxicity of the repurposed drugs in cell and animal models.

CD33 is a transmembrane receptor mainly expressed in myeloid lineage cells, especially in most leukemic blast cells, so it was a drug target for the treatment of AML [39]. In brain, it is mainly expressed on the surface of microglia. It may constitutively repress monocyte-derived pro-inflammatory cytokines [40]. The *CD33* rs3865444 risk C-allele was associated with increased CD33 expression, decreased Aβ_42_ uptake and an increased number of activated microglia that fail to clear the amyloid plaques in AD patients [41]. Hence, it might be worthwhile to explore if the repurposing of anti-CD33 antibodies/inhibitors developed for treating acute myelogenous leukemia (Gemtuzumab ozogamicin, Vadastuximab talirine, Lintuzumab, BI-836858, HuM195/rGel and HuM-195-Ac-225) are also effective for AD. Notably, Gemtuzumab ozogamicin carries a toxic calicheamicin-g1 derivative that may cause severe side effects in some patients of AML and was withdrawn from the US market in 2010 but is still on the market in Japan on the basis of a marginally favorable risk-benefit assessment [42]. This example speaks to the need of careful patient selection (e.g., based on the AD risk allele in *CD33*). Also, it is anticipated that an optimization of antibody conjugates would be needed before the benefit of anti-CD33 antibodies could be explored in AD clinical trials. Of note, another anti-CD33 antibody (Lintuzumab) was proved to be safe in human [43].

MIF is the second top ranking anti-AD target that was previously found to be elevated in the CSF of AD patients [44, 45]. As a pro-inflammatory cytokine, MIF is essential for promoting microglial activation [46]. An MIF receptor (CD74) was also documented to be elevated in microglia of AD cases [47]. Importantly, MIF interacts with Aβ and the inhibition of MIF was shown to reduce Aβ-induced toxicity in cells [45]. Therefore, existing anti-MIF antibodies might be repurposed for treating AD. If the anti-MIF antibody (clinicaltrial.gov identifier: NCT01765790) in phase I clinical trial turns out to be safe for humans, another clinical trial may be initiated to test its efficiency in AD patients with elevated MIF levels in CSF.

In addition to *CD33* and *CD74* [40, 47], other AD genes (*ABCA7* and *TREM2*) are also expressed in microglia [48, 49], suggesting that the modulation of microglial function may be a promising mechanism-based strategy for AD intervention. An immune checkpoint protein (PD-1) plays an important role in down regulating the immune system. Recently, a PD-1 immune checkpoint blockade was shown to reduce pathology and improve memory in AD mouse models [50], suggesting that PD-1 blocker drugs (e.g., Nivolumab and Pembrolizumab) might be repurposed as AD therapies.

Integrated analysis of AD-related ‘omics’ data and electronic health records would be required for better understanding AD pathogenesis and facilitating anti-AD drug repositioning. Also other databases of references, substances and reactions in chemistry (such as SciFinder) could be utilized to improve the current drug repositioning strategy. Quantitative approaches need to be developed to evaluate changes in (i) biomarkers, (ii) the effect size of risk factors, (iii) the strength of the disease-drug association, and (iv) the confidence in AD pathogenesis data. Meanwhile, integrated machine learning algorithms (such as those implemented in the IBM Watson Discovery Advisor) can be developed to automatically conduct text mining, data extraction, and computational analyses to guide the selection of drugs and their targets. Finally, it is clear that personalized medication is the future, given the high heterogeneity found in AD patients (even in subjects carrying the same genetic mutations [51, 52]).

In conclusion, systematic analyses of ‘omics’ data revealed 18 protein targets linked to 75 existing drugs, including 7 drugs inhibiting a known anti-AD target (acetylcholinesterase) that may be repurposed for treating the cognitive symptoms of AD. CD33 and MIF emerged in this analysis as particularly strong candidates on the basis of high target scores and the availability of seven existing drugs. Thus, our data has added to an increasing body of research identifying the modulation of the immune system and neuroinflammation as a promising area for anti-AD drug development. Finally, the current study highlighted the CAD protein and its link to disturbances in glutamate/GABA neurotransmitters; as well as 8 AD-related proteins detected by two ‘omics’ approaches as promising for anti-AD drug development.

## Supporting Information

S1 Table GWAS studies revealed genetic variations associated to AD with p<1.0 x 10^-5^. (PDF)

S2 Table Epigenetic studies revealed genes with significantly (p<0.05) altered epigenetic modifications related to Alzheimer's disease (AD). (PDF)

S3 Table Proteins that were significantly altered in AD patients (p<0.05). (PDF)

S4 Table AD associated metabolites retrieved from the HMDB database. (PDF)

S5 Table AD related proteins from two platforms. (PDF)

S6 Table Potential anti-AD drug targets with existing approved or clinical trial drugs. (PDF)

S7 Table Ranking of the potential AD drug targets. Target score was calculated using weighted values of 0.33, 0.34, 0.33 for fold change/odds ratio, number of citations for the paper reporting the pathogenic mechanism, and number of reports on AD and target gene, respectively. Toppgene and Toppnet scores were estimated to validate our ranking algorithm.
(PDF)
