# Supplementary material for: Drug Repositioning for Alzheimer’s Disease Based on Systematic ‘omics’ Data Mining
Source: PLoS One. 2016 Dec 22;11(12):e0168812. doi: 10.1371/journal.pone.0168812 (PMC5179106; doi:10.1371/journal.pone.0168812)
Supplement: S1 Table — (PDF) [file pone.0168812.s001.pdf]

S1 Table. GWAS studies revealed genetic variations associated to AD with $p<1.0 \times 10^{-5}$.

PUBMED ID	Initial Sample Size		Replication Sample Size		Reported Gene(s)	SNPS	P-VALUE	OR or Beta
	Cases	Controls	Cases	Controls				
20885792	931	1,104	1,338	2,003	intergenic	rs4676049	4.00E-08	1.76
22159054	513	496	NA	NA	intergenic	rs340849	8.00E-06	1.69
22159054	513	496	NA	NA	intergenic	rs11889338	9.00E-06	1.55
22159054	513	496	NA	NA	intergenic	rs2221154	3.00E-06	1.75
22159054	513	496	NA	NA	CNTNAP2	rs10273775	9.00E-06	1.52
22159054	513	496	NA	NA	intergenic	rs956225	9.00E-06	3.33
22159054	513	496	NA	NA	intergenic	rs10850408	9.00E-07	1.59
22159054	513	496	NA	NA	intergenic	rs17511627	5.00E-06	1.75
22159054	513	496	NA	NA	STK24	rs912330	4.00E-06	1.85
25778476	7,184	26,968	718	1,699	KCNN3	rs16830122	2.00E-06	1.17
25778476	7,184	26,968	718	1,699	**BIN1**	rs4663105	2.00E-12	1.19
25778476	7,184	26,968	718	1,699	SOX14, CLDN18	rs16847609	5.00E-07	1.19
25778476	7,184	26,968	718	1,699	PGBD3P4, KCTD8	rs13146780	3.00E-06	1.23
25778476	7,184	26,968	718	1,699	RAPGEF6	rs6890695	6.00E-07	1.15
25778476	7,184	26,968	718	1,699	FNIP1	rs758324	4.00E-07	1.16
25778476	7,184	26,968	718	1,699	FNIP1, ACSL6	rs142958719	3.00E-07	1.16
25778476	7,184	26,968	718	1,699	NPVF, UBA52P1	rs10807828	7.00E-06	1.10
25778476	7,184	26,968	718	1,699	MKI67, MGMT	rs61310603	8.00E-06	1.19
25778476	7,184	26,968	718	1,699	**PICALM**	rs3851179	2.00E-06	1.12
25778476	7,184	26,968	718	1,699	MACROD2, PPIAP17	rs6110969	3.00E-06	1.20
25778476	10,352	9,207	1,250	536	**CR1**	rs679515	4.00E-09	1.22
25778476	10,352	9,207	1,250	536	**BIN1**	rs6733839	1.00E-09	1.20
25778476	10,352	9,207	1,250	536	DAPL1, OR7E89P	rs1125355	4.00E-06	1.16
25778476	10,352	9,207	1,250	536	PDE1A	rs17366218	2.00E-06	1.54
25778476	10,352	9,207	1,250	536	CPS1, RPS27P10	rs178585	4.00E-06	1.13
25778476	10,352	9,207	1,250	536	CADM2	rs71316816	9.00E-06	1.25
25778476	10,352	9,207	1,250	536	LINC00290, MGC45800	rs10018288	4.00E-06	1.16
25778476	10,352	9,207	1,250	536	OFCC1, RPL7AP36	rs2876189	6.00E-06	1.14
25778476	10,352	9,207	1,250	536	RPL3P8, IMMP2L	rs59406683	6.00E-06	1.20
25778476	10,352	9,207	1,250	536	**PICALM**	rs639012	1.00E-07	1.16
25778476	10,352	9,207	1,250	536	**PICALM**	rs471470	3.00E-08	1.16
25778476	10,352	9,207	1,250	536	VN2R20P, RPL13AP7	rs721146	4.00E-06	1.17
25778476	7,184	26,968	718	1,699	**PTK2B**	rs2271920	8.00E-06	1.11
25778476	10,352	9,207	1,250	536	**PICALM**	rs10792832	4.00E-06	1.14
25778476	7,184	26,968	718	1,699	**SORL1**	rs11218343	9.00E-06	1.30
17553421	446	290	415	260	GAB2	rs2373115	1.00E-10	4.06
18823527	1,082	1,239	NA	1,400	intergenic	rs727153	3.00E-06	1.63

19136949	844	1,255	1,547	1,209	PCDH11X	rs2573905	2.00E-07	1.29
19734903	2,032	5,328	3,978	3,297	**CR1**	rs6656401, rs3818361	3.00E-10	1.22
22832961	1,291	938	2,727	3,336	intergenic	rs6856768	5.00E-06	1.18
22832961	1,291	938	2,727	3,336	intergenic	rs1357692	9.00E-06	1.16
20197096*	173 AD; 361 MCI	208	NA	NA	intergenic	rs9832461	4.00E-06	3785.6
20197096*	173 AD; 361 MCI	208	NA	NA	intergenic	rs1448284	2.00E-06	9939.9
20197096*	173 AD; 361 MCI	208	NA	NA	KIAA0743	rs7155434	8.00E-06	3643.0
23535033	303	NA	323	NA	PRRC2C	rs2421847	9.00E-07	0.26
23535033	303	NA	323	NA	FMN2	rs12091371	7.00E-08	0.17
23535033	303	NA	323	NA	CTNNA2	rs6738962	1.00E-08	0.18
23535033	303	NA	323	NA	LIMS2	rs78022502	2.00E-06	0.23
23535033	303	NA	323	NA	STK32B	rs78647349	5.00E-07	0.30
23535033	303	NA	323	NA	AFF1	rs340635	2.00E-07	0.23
23535033	303	NA	323	NA	ANKRD55	rs4700060	1.00E-08	0.21
23535033	303	NA	323	NA	PGAM5P1, MAN2A1	rs112724034	9.00E-13	0.31
23535033	303	NA	323	NA	CAMK4	rs77636885	2.00E-06	0.30
23535033	303	NA	323	NA	DMXL1	rs116348108	9.00E-07	0.28
23535033	303	NA	323	NA	MEGF10	rs143954261	8.00E-07	0.29
23535033	303	NA	323	NA	FLJ33630	rs146579248	4.00E-07	0.21
23535033	303	NA	323	NA	SAP30L	rs148763909	1.00E-08	0.15
23535033	303	NA	323	NA	NKAIN2	rs117780815	6.00E-07	0.31
23535033	303	NA	323	NA	PDE7B	rs11154851	1.00E-08	0.25
23535033	303	NA	323	NA	PLEKHG1	rs75253868	2.00E-06	0.26
23535033	303	NA	323	NA	CYCS	rs1861525	2.00E-07	0.25
23535033	303	NA	323	NA	ELMO1	rs2392492	1.00E-06	0.32
23535033	303	NA	323	NA	HECW1	rs17172199	1.00E-06	0.28
23535033	303	NA	323	NA	EXOC4	rs11770757	5.00E-07	0.16
23535033	303	NA	323	NA	CSMD1	rs73660619	8.00E-07	0.26
23535033	303	NA	323	NA	ST18	rs7009219	5.00E-07	0.16
23535033	303	NA	323	NA	NCS1	rs4836694	7.00E-07	0.21
23535033	303	NA	323	NA	PPAPDC1A	rs118048115	6.00E-07	0.34
23535033	303	NA	323	NA	RRAS2, PSMA1, SPON1	rs11023139	7.00E-11	0.31
23535033	303	NA	323	NA	ARHGAP20	rs326946	7.00E-07	0.16
23535033	303	NA	323	NA	SLC4A8	rs147845115	3.00E-07	0.29

23535033	303	NA	323	NA	CRADD	rs61144803	5.00E-08	0.16
23535033	303	NA	323	NA	ANO4	rs1399439	4.00E-07	0.20
23535033	303	NA	323	NA	GPC6	rs143258881	7.00E-08	0.29
23535033	303	NA	323	NA	MYO16	rs17393344	2.00E-08	0.26
23535033	303	NA	323	NA	CLMN	rs115102486	2.00E-08	0.31
23535033	303	NA	323	NA	GABRG3	rs74006954	3.00E-07	0.28
23535033	303	NA	323	NA	LIPC	rs17301739	2.00E-06	0.28
23535033	303	NA	323	NA	FLJ45256	rs8045064	4.00E-08	0.21
23535033	303	NA	323	NA	VAT1L	rs9934540	4.00E-07	0.25
23535033	303	NA	323	NA	BCAS3	rs72832584	1.00E-11	0.30
23535033	303	NA	323	NA	TGM6	rs34972666	3.00E-08	0.23
23535033	303	NA	323	NA	PARVB	rs75617873	5.00E-07	0.17
24755620	2,540	2,029	NA	NA	intergenic	rs13178362	7.00E-07	1.33
25649651	983	NA	NA	NA	NCKAP5	rs7588354	1.00E-06	1.52
25649651	983	NA	NA	NA	PKNOX2	rs11601321	8.00E-06	1.63
20885792	931	1,104	1,338	2,003	**APOE**, TOMM40	rs2075650	5.00E-36	2.94
20885792	931	1,104	1,338	2,003	MTHFD1L	rs11754661	2.00E-10	2.10
20885792	931	1,104	1,338	2,003	PVRL2	rs6859	1.00E-07	1.41
22159054	513	496	NA	NA	SLC4A1AP	rs17006206	2.00E-06	2.05
22159054	513	496	NA	NA	POLN	rs1923775	6.00E-06	1.60
22159054	513	496	NA	NA	PVRL2	rs6859	5.00E-07	1.58
20460622	3006	22,604	6,505	13,532	**APOE**	rs2075650	1.00E-295	2.53
23571587	1,968	3,928	NA	NA	**ABCA7**, HMHA1, GRIN3B	rs115550680	2.00E-09	1.79
23571587	1,968	3,928	NA	NA	intergenic	rs145848414	7.00E-08	2.29
25778476	17,536	36,175	1,968	2,235	IRF6	rs59043219	9.00E-06	1.19
25778476	17,536	36,175	1,968	2,235	GAPDHP15, RBBP4P4	rs1936246	2.00E-13	1.04
25778476	17,536	36,175	1,968	2,235	PMS2CL, ZNF12	rs116139393	3.00E-08	1.10
25778476	17,536	36,175	1,968	2,235	THSD7A, TMEM106B	rs1595014	4.00E-06	1.19
25778476	7,184	26,968	718	1,699	IL21, CETN4P	rs78538083	8.00E-06	1.32
25778476	7,184	26,968	718	1,699	SERINC5, KRT18P45	rs71636213	9.00E-06	1.18
25778476	7,184	26,968	718	1,699	CDC42SE2	rs382216	2.00E-07	1.15
25778476	7,184	26,968	718	1,699	ACSL6	rs476428	2.00E-07	1.16
25778476	7,184	26,968	718	1,699	PFDN1, HBEGF	rs11168036	3.00E-07	1.12
25778476	7,184	26,968	718	1,699	**EPHA1,** TAS2R62P	rs75045569	8.00E-06	1.15
25778476	7,184	26,968	718	1,699	**CLU**	rs2279590	2.00E-06	1.12
25778476	7,184	26,968	718	1,699	USP6NL, ECHDC3	rs7920721	3.00E-06	1.12
25778476	7,184	26,968	718	1,699	SH2D4B	rs59616746	1.00E-06	1.30
25778476	7,184	26,968	718	1,699	MS4, **MS4A6A**, MS4A4A, MS4A6E	rs1582763	2.00E-09	1.15
25778476	7,184	26,968	718	1,699	**SLC24A4**	rs10498633	4.00E-07	1.15
25778476	7,184	26,968	718	1,699	CRHR1	rs7207400	5.00E-06	1.14

25778476	7,184	26,968	718	1,699	WNT3	rs199499	6.00E-06	1.16
25778476	7,184	26,968	718	1,699	C17orf28, CDR2L	rs71380849	9.00E-07	1.47
25778476	7,184	26,968	1,250	536	FBXO40	rs9869689	3.00E-06	1.21
25778476	7,184	26,968	1,250	536	PEX6	rs1129187	7.00E-06	1.13
25778476	7,184	26,968	1,250	536	**CLU**	rs9331896	3.00E-09	1.19
25778476	7,184	26,968	1,250	536	SORD	rs2854437	2.00E-06	1.23
17474819	664	422	NA	NA	**APOE**	rs4420638	1.00E-39	4.01
19734902	3,941	7,848	2,023	2,340	**CLU**	rs11136000	9.00E-10	1.16
19734902	3,941	7,848	2,023	2,340	**PICALM**	rs3851179	1.00E-09	1.16
19734903	2,032	5,328	3,978	3,297	**CLU**	rs2279590, rs11136000, rs9331888	6.00E-10	1.22
19734902	3,941	7,848	2,023	2,340	**APOE,** TOMM40	rs2075650	2.00E-157	2.53
21379329	1,848	1,991	617	573	CUGBP2	rs62209	2.00E-07	2.04
21390209	1,831	1,764	751	751	**BIN1**	rs744373	1.00E-10	1.19
21390209	1,831	1,764	751	751	**BIN1**	rs12989701	3.00E-10	1.23
21460840	6,688	13,685	13,182	26,161	**ABCA7**	rs3764650	5.00E-17	1.23
21460840	6,688	13,685	13,182	26,161	**MS4A4E, MS4A6A**	rs610932	2.00E-14	1.11
21460841	8,309	7,366	10,523	28,231	**CD2AP**	rs9349407	9.00E-09	1.11
21460841	8,309	7,366	10,523	28,231	**EPHA1**	rs11767557	6.00E-10	1.11
21460841	8,309	7,366	10,523	28,231	MS4A4A	rs4938933	8.00E-12	1.12
21460841	8,309	7,366	10,523	28,231	**CD33**	rs3865444	2.00E-09	1.10
21460840	6,688	13,685	13,182	26,161	**CR1**	rs3818361	4.00E-14	1.18
21460840	6,688	13,685	13,182	26,161	**BIN1**	rs744373	3.00E-14	1.17
21460841	8,309	7,366	10,523	28,231	**CR1**	rs6701713	5.00E-10	1.16
21460841	8,309	7,366	10,523	28,231	**BIN1**	rs7561528	4.00E-14	1.17
21460841	8,309	7,366	10,523	28,231	**CLU**	rs1532278	8.00E-08	1.12
21460841	8,309	7,366	10,523	28,231	**PICALM**	rs561655	7.00E-11	1.15
21460841	8,309	7,366	10,523	28,231	**ABCA7**	rs3752246	6.00E-07	1.15
21627779	3,009	3,006	7,172	11,335	MS4A	rs1562990	4.00E-11	1.14
21627779	3,009	3,006	7172	11335	**APOE**	rs157580	8.00E-89	1.69
21627779	3,009	3,006	7172	11335	**PICALM**	rs536841	3.00E-09	1.16
21627779	3,009	3,006	7172	11335	**CLU**	rs569214	4.00E-08	1.14
21627779	3,009	3,006	7172	11335	**BIN1**	rs744373	2.00E-09	1.18
22430674	2,025	5,328	7,913	10,417	FRMD4A	rs7081208, rs2446581, rs17314229	1.00E-10	1.68
22832961	1,291	938	2,727	3,336	**APOE**, TOMM40, APOC1	rs4420638	8.00E-149	3.45
22832961	1,291	938	2,727	3,336	**BIN1**	rs7561528	6.00E-11	1.25
22832961	1,291	938	2,727	3,336	**PICALM**	rs17817600	2.00E-08	1.33
22832961	1,291	938	2,727	3,336	PPP1R3B	rs3748140	3.00E-07	2.43
22832961	1,291	938	2,727	3,336	MMP3, MMP12	rs12808148	1.00E-06	1.23

22832961	1,291	938	2,727	3,336	FLJ37543	rs11738335	5.00E-06	1.20
23374588	92	77	94	74	SLC9A9	rs17636071	2.00E-06	4.74
23374588	92	77	94	74	EFR3A	rs2270875	9.00E-06	2.84
23374588	92	77	94	74	intergenic	rs17798800	7.00E-06	2.63
22005931	2,222	NA	NA	NA	intergenic	rs271066	3.00E-06	0.65
22005931	2,222	NA	NA	NA	ADAMTS9	rs704454	3.00E-06	0.50
22005931	2,222	NA	NA	NA	DCHS2	rs1466662	5.00E-07	0.96
22005931	2,222	NA	NA	NA	KCNV2, VLDLR	rs2034764	4.00E-06	0.88
22005931	2,222	NA	NA	NA	HRK, RNFT2	rs17429217	3.00E-06	2.44
22005931	2,222	NA	NA	NA	SLC28A1, ZNF592, ALPK3	rs3743162	9.00E-06	0.88
22005931	2,222	NA	NA	NA	**APOE**, TOMM40, APOC1, PVRL2	rs4420638	1.00E-12	2.20
22005931	2,222	NA	NA	NA	intergenic	rs10517270	4.00E-06	3.31
22005931	2,222	NA	NA	NA	LEMD2, MLN, MIR1275	rs2104362	7.00E-06	0.84
22005931	2,222	NA	NA	NA	intergenic	rs12933233	8.00E-06	0.79
22005931	2,222	NA	NA	NA	LOC390958, Sec11C	rs1037757	8.00E-06	4.56
22005931	2,222	NA	NA	NA	ZNF592, ALPK3, SLC28A1	rs3743162	9.00E-06	0.88
22005931	2,222	NA	NA	NA	intergenic	rs12816806	9.00E-06	0.84
22005930	1,299	5,659	NA	NA	RP11-242G20.2	rs4746003	6.00E-06	1.30
22005930	1,299	5,659	NA	NA	RP11-291J9.2	rs4576506	1.00E-06	1.66
22005930	1,299	5,659	NA	NA	SLC2A9	rs733175	5.00E-06	1.37
22005930	1,299	5,659	NA	NA	AP003097.1	rs10792830	6.00E-06	1.25
22005930	1,299	5,659	NA	NA	AC015804.1	rs16970672	2.00E-06	1.29
22005930	1,299	5,659	NA	NA	VSNL1	rs4038131	6.00E-07	1.54
22005930	1,299	5,659	NA	NA	**BIN1**	rs10207628	1.00E-06	1.41
22005930	1,299	5,659	NA	NA	RP11-572M11.4	rs9811423	4.00E-06	1.28
22005930	1,299	5,659	NA	NA	RIMBP2	rs1464108	8.00E-06	1.28
22005930	1,299	5,659	NA	NA	MPP7	rs11006923	9.00E-06	1.59
22005930	1,299	5,659	NA	NA	**APOE**	rs157582	9.00E-52	2.30
20197096*	173 AD; 361 MCI	208	NA	NA	GRIN2B	rs11055612	3.00E-06	3371.60
20197096*	173 AD; 361 MCI	208	NA	NA	intergenic	rs2456930	3.00E-07	3843.90
23150908	3,550	8,888	2,037	9,727	**TREM2**	rs75932628	2.00E-12	2.90
24162737	17,008	37,154	8,572	11,312	**CR1**	rs6656401	6.00E-24	1.18
24162737	17,008	37,154	8,572	11,312	**BIN1**	rs6733839	7.00E-44	1.22
24162737	17,008	37,154	8,572	11,312	**CD2AP**	rs10948363	5.00E-11	1.10
24162737	17,008	37,154	8,572	11,312	**EPHA1**	rs11771145	1.00E-13	1.11

24162737	17,008	37,154	8,572	11,312	**CLU**	rs9331896	3.00E-25	1.16
24162737	17,008	37,154	8,572	11,312	**MS4A6A**	rs983392	6.00E-16	1.11
24162737	17,008	37,154	8,572	11,312	**PICALM**	rs10792832	9.00E-26	1.15
24162737	17,008	37,154	8,572	11,312	**INPP5D**	rs35349669	3.00E-08	1.08
24162737	17,008	37,154	8,572	11,312	**MEF2C**	rs190982	3.00E-08	1.08
24162737	17,008	37,154	8,572	11,312	**NME8**	rs2718058	5.00E-09	1.08
24162737	17,008	37,154	8,572	11,312	**CELF1**	rs10838725	1.00E-08	1.08
24162737	17,008	37,154	8,572	11,312	**FERMT2**	rs17125944	8.00E-09	1.14
24162737	17,008	37,154	8,572	11,312	**CASS4**	rs7274581	3.00E-08	1.14
24162737	17,008	37,154	8,572	11,312	**PTK2B**	rs28834970	7.00E-14	1.10
24162737	17,008	37,154	8,572	11,312	**SORL1**	rs11218343	1.00E-14	1.30
24162737	17,008	37,154	8,572	11,312	**SLC24A4, RIN3**	rs10498633	6.00E-09	1.10
24162737	17,008	37,154	8,572	11,312	intergenic	rs6678275	3.00E-07	1.09
24162737	17,008	37,154	8,572	11,312	HS3ST1	rs6448799	7.00E-08	1.08
24162737	17,008	37,154	8,572	11,312	SQSTM1	rs72807343	7.00E-07	1.35
24162737	17,008	37,154	8,572	11,312	TREML2	rs9381040	6.00E-07	1.08
24162737	17,008	37,154	8,572	11,312	NDUFAF6	rs7818382	8.00E-08	1.07
24162737	17,008	37,154	8,572	11,312	ECHDC3	rs7920721	3.00E-07	1.07
24162737	17,008	37,154	8,572	11,312	ADAMTS20	rs7295246	3.00E-07	1.07
24162737	17,008	37,154	8,572	11,312	SPPL2A	rs8035452	3.00E-07	1.08
24162737	17,008	37,154	8,572	11,312	TRIP4	rs74615166	4.00E-07	1.29
24162737	17,008	37,154	8,572	11,312	SCIMP	rs7225151	4.00E-07	1.10
24162737	17,008	37,154	8,572	11,312	ACE	chr17:61538148	3.00E-07	1.34
24162737	17,008	37,154	8,572	11,312	**CD33**	rs3865444	3.00E-06	1.06
23565137	891	844	13,064	13,045	**SORL1**	rs11218343	2.00E-09	1.23
23562540	591	687	NA	NA	**APOE**, TOMM40	rs769449	2.00E-16	0.08
23562540	591	687	NA	NA	**APOE**, TOMM40	rs769449	2.00E-18	0.09
23562540	591	687	NA	NA	UTS2D, SNAR-I, GEMC1, OSTNIL1-RAP, CCDC50	rs9877502	5.00E-09	0.05
23562540	591	687	NA	NA	GLIS3	rs514716	1.00E-08	0.07
23562540	591	687	NA	NA	GLIS3	rs514716	3.00E-09	0.07
23562540	591	687	NA	NA	TREM, NCR2	rs6922617	4.00E-08	0.09
23535033	303	NA	323	NA	MOBP	rs538867	1.00E-07	0.26
23535033	303	NA	323	NA	BZW2, TSPAN13	rs58370486	6.00E-11	0.36
23535033	303	NA	323	NA	OSBPL7, SP6, MRPL10	rs4794202	8.00E-08	0.19
23535033	303	NA	323	NA	CACNA1G	rs117964204	1.00E-09	0.28
23535033	303	NA	323	NA	LOC390956	rs7245858	2.00E-06	0.28
24755620	2,540	2,029	NA	NA	**APOE**, BCAM, PVR, EXOC3L2, TOMM40, PVRL2, BCL3, APOC1	rs2075650	9.00E-116	4.48

24755620	2,540	2,029	NA	NA	intergenic	rs249153	4.00E-07	1.41
24755620	2,540	2,029	NA	NA	MS4A3	rs474951	1.00E-06	1.27
24755620	2,540	2,029	NA	NA	FANCD2, FANCD2OS	rs1552244	2.00E-06	1.32
25027320	363	NA	515	NA	SUCLG2	rs62256378	3.00E-12	0.71
22005930	2,034	NA	NA	NA	AC110611.1	rs753129	3.00E-07	1.52
22005930	2,034	NA	NA	NA	AC008541.1	rs257016	4.00E-06	1.43
22005930	2,034	NA	NA	NA	DIP2C	rs11252926	8.00E-06	1.39
22005930	2,034	NA	NA	NA	AC079250.1	rs2969775	2.00E-06	1.47
22005930	2,034	NA	NA	NA	RP11-341A22.2	rs16922670	7.00E-06	1.63
22005930	2,034	NA	NA	NA	NR	rs17716202	8.00E-06	2.22
25649651	983	NA	NA	NA	IL19	rs2243170	7.00E-07	1.65
25649651	983	NA	NA	NA	CCDC85C	rs2400749	2.00E-06	1.39
25649651	983	NA	NA	NA	SDR9C7	rs840163	2.00E-06	1.40
25649651	983	NA	NA	NA	NARS2, GAB2	rs4474465	3.00E-06	1.44
25649651	983	NA	NA	NA	ALDH4A1	rs6695033	3.00E-06	2.71
21116278*	236 AD; 424 MCI	279	NA	NA	TUSC1	rs17774966	8.00E-06	0.15
21116278*	236 AD; 424 MCI	279	NA	NA	ZNF292	rs1925690	3.00E-08	3.00E-04
21116278*	236 AD; 424 MCI	279	NA	NA	CDC42EP3	rs4670766	2.00E-06	0.005
21116278*	236 AD; 424 MCI	279	NA	NA	UTS2D	rs10937470	9.00E-06	0.004
21116278*	236 AD; 424 MCI	279	NA	NA	PAK2	rs2084385	5.00E-06	0.006
21116278*	236 AD; 424 MCI	279	NA	NA	ZPBP	rs7805803	9.00E-06	0.004
21116278*	236 AD; 424 MCI	279	NA	NA	ADCY8	rs263238	2.00E-06	0.006
21116278*	236 AD; 424 MCI	279	NA	NA	LOC392298	rs12555345	5.00E-06	0.004
21116278*	236 AD; 424	279	NA	NA	PCSK5	rs10512049	3.00E-06	0.007

	MCI							
21116278*	236 AD; 424 MCI	279	NA	NA	PDS5B	rs990324	5.00E-06	0.006
21116278*	236 AD; 424 MCI	279	NA	NA	EDNRB, POU4F1	rs9574199	7.00E-06	0.004
21116278*	236 AD; 424 MCI	279	NA	NA	FARP1	rs4318070	7.00E-06	0.004
21116278*	236 AD; 424 MCI	279	NA	NA	RORA	rs3784609	3.00E-06	0.005
21116278*	245 AD; 434 MCI	284	NA	NA	SLC44A5	rs1857353	2.00E-06	0.016
21116278*	245 AD; 434 MCI	284	NA	NA	TXNDC6	rs9846480	8.00E-06	0.009
21116278*	245 AD; 434 MCI	284	NA	NA	ABCA1	rs3905000	9.00E-06	0.013
21116278*	245 AD; 434 MCI	284	NA	NA	YAP1, BIRC3	rs2852894	1.00E-06	0.016
25778476	7,184	26,968	718	1,699	FAM20C	rs11761441	2.00E-06	1.12
25778476	7,184	26,968	718	1,699	KANSL1, LRRC27A, MAPT	rs2732703	6.00E-09	1.37
24162737	17,008	37,154	8,572	11,312	**ABCA7**	rs4147929	1.00E-15	1.15
24162737	17,008	37,154	8,572	11,312	**ZCWPW1**	rs1476679	6.00E-10	1.10
24162737	17,008	37,154	8,572	11,312	AP2A2	rs10751667	6.00E-07	1.08
23565137	891	844	13,064	13,045	**APOE,** CEACAM16, BCL3, PVRL2, TOMM40, PPP1R37	rs519113	5.00E-39	2.09
25027320	363	NA	515	NA	**APOE**	rs429358	4.00E-17	0.40
22005930	2,034	NA	NA	NA	ZNF320	rs6509701	5.00E-06	1.41
22005930	2,034	NA	NA	NA	STK11	rs3764640	8.00E-06	1.47
24162737	17,008	37,154	8,572	11,312	**HLA-DRB5, HLA-DRB1**	rs9271192	3.00E-12	1.11
24162737	17,008	37,154	8,572	11,312	IGH	rs2337406	3.00E-07	1.15

NA: not available

Bold: well confirmed AD genes

* Denotes studies with the beta coefficient
