# Supplementary material for: Drug Repositioning for Alzheimer’s Disease Based on Systematic ‘omics’ Data Mining
Source: PLoS One. 2016 Dec 22;11(12):e0168812. doi: 10.1371/journal.pone.0168812 (PMC5179106; doi:10.1371/journal.pone.0168812)
Supplement: S2 Table — (PDF) [file pone.0168812.s002.pdf]

S2 Table. Epigenetic studies revealed genes with significantly ($p<0.05$) altered epigenetic modifications related to Alzheimer's disease (AD).

Protein ID	Gene	Source	Sample size	Tissues	Platform	PUBMED ID
P16157	ANK1	human	708	Dorsolateral prefrontal cortex	Illumina 450K	PMID: 25129075
Q9H251	CDH23	human	708	Dorsolateral prefrontal cortex	Illumina 450K	PMID: 25129075
Q14689	DIP2A	human	708	Dorsolateral prefrontal cortex	Illumina 450K	PMID: 25129075
Q6PJF5	RHBDF2	human	708	Dorsolateral prefrontal cortex	Illumina 450K	PMID: 25129075
P26373	RPL13	human	708	Dorsolateral prefrontal cortex	Illumina 450K	PMID: 25129075
P36955	SERPINF1	human	708	Dorsolateral prefrontal cortex	Illumina 450K	PMID: 25129075
P08697	SERPINF2	human	708	Dorsolateral prefrontal cortex	Illumina 450K	PMID: 25129075
Q8IZY2	ABCA7	human	708	Dorsolateral prefrontal cortex	Illumina 450K	PMID: 25129075
O00499	BIN1	human	708	Dorsolateral prefrontal cortex	Illumina 450K	PMID: 25129075
Q9BXS4	TMEM59	human	12	Frontal Cortex	Illumina HM27	PMID: 22451312
P16157	ANK1	human	328	Brain	Illumina 450K	PMID: 25129077
Q13492	PICALM	mouse	36	Hippocampus	Chip-seq and RNA-seq	PMID: 25693568
O00499	BIN1	mouse	36	Hippocampus	Chip-seq and RNA-seq	PMID: 25693568
Q92835	INPP5D	mouse	36	Hippocampus	Chip-seq and RNA-seq	PMID: 25693568
Q92879	CELF1	mouse	36	Hippocampus	Chip-seq and RNA-seq	PMID: 25693568
P17947	SPI1	mouse	36	Hippocampus	Chip-seq and RNA-seq	PMID: 25693568
