# Supplementary material for: Drug Repositioning for Alzheimer’s Disease Based on Systematic ‘omics’ Data Mining
Source: PLoS One. 2016 Dec 22;11(12):e0168812. doi: 10.1371/journal.pone.0168812 (PMC5179106; doi:10.1371/journal.pone.0168812)
Supplement: S3 Table — (PDF) [file pone.0168812.s003.pdf]

S3 Table. Proteins that were significantly altered in AD patients (p<0.05).

Protein name	Gene name	Uniprot ID	Sample	Sample size		Platform	PUBMED ID
				Control	AD		
PKCG	PKCG	P05129	Brain - cortex	10	10	Label-Free Quantitative LC-MS/WB	PMC3445701
NUMBL	NUMBL	Q9Y6R0	Brain - cortex	10	10	Label-Free Quantitative LC-MS/WB	PMC3445701
Amyloid Protein Precursor	APP	P05067	CSF	31	32	ICAT-MS/WB	PMID: 15851850
Cathepsin B Precursor	CTSB	P07858	CSF	31	32	ICAT-MS/WB	PMID: 15851850
proSAAS	proSAAS	Q9UHG2	CSF	3	3	MRM/ELISA/Multiple x	PMC3710693
ApoE	APOE	P02649	CSF	3	3	MRM/ELISA/Multiple x	PMC3710693
TTR	TTR	P02766	CSF	3	3	MRM/ELISA/Multiple x	PMC3710693
fibrin beta	FGB	P02675	CSF	3	3	MRM/ELISA/Multiple x	PMC3710693
VDBP	VDBP	Q6LDC6	CSF	3	3	MRM/ELISA/Multiple x	PMC3710693
Abeta 42	APP	P05067	CSF	242	91	Multiplexed immunoassay	PMC3079734
Tau	MAPT	P10636	CSF	242	91	Multiplexed immunoassay	PMC3079734
Growth regulated alpha protein	GRO	P09341	CSF	242	91	Multiplexed immunoassay	PMC3079734
matrix metalloproteinase-10	MMP-10	P09238	CSF	242	91	Multiplexed immunoassay	PMC3079734
plasminogen activator inhibitor1	PAI-1	P05121	CSF	242	91	Multiplexed immunoassay	PMC3079734
TNF-related apoptosis-inducing ligand receptor 3	TRAIL-R3	O14798	CSF	242	91	Multiplexed immunoassay	PMC3079734
Vascular endothelial growth factor	VEGF	P15692	CSF	242	91	Multiplexed immunoassay	PMC3079734
pancreatic polypeptide	PPY	P01298	CSF	242	91	Multiplexed immunoassay	PMC3079734
FAS	FAS	P25445	CSF	242	91	Multiplexed immunoassay	PMC3079734
macrophage migration inhibitory factor	MIF	P14174	CSF	242	91	Multiplexed immunoassay	PMC3079734
interleukin-7	IL-7	P13232	CSF	242	91	Multiplexed immunoassay	PMC3079734
cystatin C	CST3	P01034	CSF	242	91	Multiplexed immunoassay	PMC3079734
thrombopoietin	THPO	P40225	CSF	242	91	Multiplexed immunoassay	PMC3079734
sortilin	SORT1	Q99523	CSF	242	91	Multiplexed immunoassay	PMC3079734

Monocyte-chemotactic protein 2	MCP-2	P80075	CSF	242	91	Multiplexed immunoassay	PMC3079734
Tymus-expressed chemokine	TECK	O15444	CSF	242	91	Multiplexed immunoassay	PMC3079734
eotaxin-3	CCL26	Q9Y258	CSF	242	91	Multiplexed immunoassay	PMC3079734
interleukin-17E	IL-17E	Q9H293	CSF	242	91	Multiplexed immunoassay	PMC3079734
kdney injury molecule-1	KIM-1	Q96D42	CSF	242	91	Multiplexed immunoassay	PMC3079734
heparin-inding epidermal growth factor-like growth factor	HB-EGF	Q99075	CSF	242	91	Multiplexed immunoassay	PMC3079734
Secreted phosphoprotein 1	SPP1	P10451	CSF	242	91	Multiplexed immunoassay	PMC3079734
alpha-1-antitrypsin	SERPINA 1	P01009	CSF	242	91	Multiplexed immunoassay	PMC3079734
IGFBP-2	IGFBP-2	P18065	CSF	242	91	Multiplexed immunoassay	PMC3079734
IL-10	IL-10	P22301	CSF	242	91	Multiplexed immunoassay	PMC3079734
TNF RII	TNF RII	P20333	CSF	242	91	Multiplexed immunoassay	PMC3079734
resistin	RETN	Q9HD89	CSF	242	91	Multiplexed immunoassay	PMC3079734
FABP	FABP	O15540	CSF	242	91	Multiplexed immunoassay	PMC3079734
APOD	APOD	P05090	CSF	242	91	Multiplexed immunoassay	PMC3079734
HGF	HGF	P14210	CSF	242	91	Multiplexed immunoassay	PMC3079734
insulin	INS	P01308	CSF	242	91	Multiplexed immunoassay	PMC3079734
IP-10	IP-10	P02778	CSF	242	91	Multiplexed immunoassay	PMC3079734
thrombomodulin	THBD	P07204	CSF	242	91	Multiplexed immunoassay	PMC3079734
SPARC	SPARC	P09486	CSF	80	80	MALDI-TOF/ELISA/WB	PMID: 22045497
Abeta 40	APP	P05067	CSF	80	80	MALDI-TOF/ELISA/WB	PMID: 22045497
Abeta 42	APP	P05067	CSF	80	80	MALDI-TOF/ELISA/WB	PMID: 22045497
Tau	MAPT	P10636	CSF	80	80	MALDI-TOF/ELISA/WB	PMID: 22045497
hemopexin	HPX	P02790	CSF	5	14	Stable isotope-LC-MS	PMID: 22232349
neuronal pentraxin receptor	NPTXR	O95502	CSF	5	14	Stable isotope-LC-MS	PMID: 22232349
Afamin precursor	AFM	P43652	CSF	5	14	Stable isotope-LC-MS	PMID: 22232349
α1-Type XVIII collagen isoform 3 precursor	COL18A1	P39060	CSF	5	14	Stable isotope-LC-MS	PMID: 22232349
α1B-Glycoprotein	A1BG	P04217	CSF	5	14	Stable isotope-LC-MS	PMID: 22232349

precursor							
α2-Macroglobulin precursor	A2M	P01023	CSF	5	14	Stable isotope-LC-MS	PMID: 22232349
Amyloid β(A4) precursorlike protein 2 isoform 4	APLP2	Q06481	CSF	5	14	Stable isotope-LC-MS	PMID: 22232349
Amyloid β A4 protein isoform g	APP	P05067	CSF	5	14	Stable isotope-LC-MS	PMID: 22232349
Apolipoprotein C-I precursor	APOC1	P02654	CSF	5	14	Stable isotope-LC-MS	PMID: 22232349
Biotinidase precursor	BTD	P43251	CSF	5	14	Stable isotope-LC-MS	PMID: 22232349
Brain neuron cytoplasmic protein 1	NSG1	P42857	CSF	5	14	Stable isotope-LC-MS	PMID: 22232349
Calsyntenin 3	CLSTN3	Q9BQT9	CSF	5	14	Stable isotope-LC-MS	PMID: 22232349
Cathepsin L1 preproprotein	CTSL1	P07711	CSF	5	14	Stable isotope-LC-MS	PMID: 22232349
CD99 antigen isoform b precursor	CD99	P14209	CSF	5	14	Stable isotope-LC-MS	PMID: 22232349
Ceruloplasmin precursor	CP	P00450	CSF	5	14	Stable isotope-LC-MS	PMID: 22232349
Di-N-acetyl-chitobiase	CTBS	Q01459	CSF	5	14	Stable isotope-LC-MS	PMID: 22232349
Complement component 2 isoform 2 preproprotein	C2	P06681	CSF	5	14	Stable isotope-LC-MS	PMID: 22232349
Complement component 4A preproprotein	C4A	P0C0L4	CSF	5	14	Stable isotope-LC-MS	PMID: 22232349
Complement component 4B preproprotein	C4B	P0C0L5	CSF	5	14	Stable isotope-LC-MS	PMID: 22232349
Complement component 5 preproprotein	C5	P01031	CSF	5	14	Stable isotope-LC-MS	PMID: 22232349
Complement component 6 precursor	C6	P13671	CSF	5	14	Stable isotope-LC-MS	PMID: 22232349
Complement factor B preproprotein	CFB	P00751	CSF	5	14	Stable isotope-LC-MS	PMID: 22232349
Enolase 2	ENO2	P09104	CSF	5	14	Stable isotope-LC-MS	PMID: 22232349
Fructose-bisphosphate aldolase A	ALDOA	P04075	CSF	5	14	Stable isotope-LC-MS	PMID: 22232349
Glutamate receptor, ionotrophic, AMPA 4 isoform 3 precursor	GLUR4	P48058	CSF	5	14	Stable isotope-LC-MS	PMID: 22232349
Hypothetical protein LOC729956 (Homo sapiens)	SHISA7	A6NL88	CSF	5	14	Stable isotope-LC-MS	PMID: 22232349
Insulinlike growth factor binding protein 6	IGFBP6	P24592	CSF	5	14	Stable isotope-LC-MS	PMID: 22232349
Inter-α (globulin) inhibitor H1	ITIH1	P19827	CSF	5	14	Stable isotope-LC-MS	PMID: 22232349

L1 cell adhesion molecule isoform 3 precursor	L1CAM	P32004	CSF	5	14	Stable isotope-LC-MS	PMID: 22232349
Laminin, β2 precursor	LAMB2	P55268	CSF	5	14	Stable isotope-LC-MS	PMID: 22232349
Latent transforming growth factor β-binding protein 2	LTBP2	Q14767	CSF	5	14	Stable isotope-LC-MS	PMID: 22232349
Leucine-rich α2-glycoprotein 1	LRG1	P02750	CSF	5	14	Stable isotope-LC-MS	PMID: 22232349
Melanoma cell adhesion molecule	MCAM	P43121	CSF	5	14	Stable isotope-LC-MS	PMID: 22232349
Neogenin homologue 1	NEO1	Q92859	CSF	5	14	Stable isotope-LC-MS	PMID: 22232349
Neuronal growth regulator 1	NEGR1	Q7Z3B1	CSF	5	14	Stable isotope-LC-MS	PMID: 22232349
Osteoglycin preproprotein	OGN	P20774	CSF	5	14	Stable isotope-LC-MS	PMID: 22232349
Peptidoglycan recognition protein 2 precursor	PGLYRP2	Q96PD5	CSF	5	14	Stable isotope-LC-MS	PMID: 22232349
Peptidylglycine α-amidating monooxygenase isoform a preproprotein	PAM	P19021	CSF	5	14	Stable isotope-LC-MS	PMID: 22232349
Plasminogen	PLG	P00747	CSF	5	14	Stable isotope-LC-MS	PMID: 22232349
Poliovirus receptor–related 1 isoform 1	PVRL1	Q15223	CSF	5	14	Stable isotope-LC-MS	PMID: 22232349
Protein disulfide-isomerase A3 precursor	PDIA3	P30101	CSF	5	14	Stable isotope-LC-MS	PMID: 22232349
Protein tyrosine phosphatase, receptor type, D isoform 5 precursor	PTPRN	Q16849	CSF	5	14	Stable isotope-LC-MS	PMID: 22232349
Protein-L-isoaspartate (d-aspartate) O-methyltransferase	PCMT1	P22061	CSF	5	14	Stable isotope-LC-MS	PMID: 22232349
Pyruvate kinase, muscle isoform M1	PKM	P14618	CSF	5	14	Stable isotope-LC-MS	PMID: 22232349
Ig γ3-chain C region	IGHG3	P01860	CSF	5	14	Stable isotope-LC-MS	PMID: 22232349
Secreted modular calcium-binding protein 1 isoform 1	SMOC1	Q9H4F8	CSF	5	14	Stable isotope-LC-MS	PMID: 22232349
Secreted phosphoprotein 1 isoform a	SPP1	P10451	CSF	5	14	Stable isotope-LC-MS	PMID: 22232349
Secreted phosphoprotein 1 isoform b	SPP1	P10451	CSF	5	14	Stable isotope-LC-MS	PMID: 22232349
Secreted phosphoprotein 1 isoform c	SPP1	P10451	CSF	5	14	Stable isotope-LC-MS	PMID: 22232349

Seizure-related 6 homologue (mouse)–like precursor	SEZ6L	Q9BYH1	CSF	5	14	Stable isotope-LC-MS	PMID: 22232349
Serpin peptidase inhibitor, clade A, member 3 precursor	SERPINA 3	P01011	CSF	5	14	Stable isotope-LC-MS	PMID: 22232349
Slitlike 2	SLITL2	Q6EMK4	CSF	5	14	Stable isotope-LC-MS	PMID: 22232349
Spondin 1, extracellular matrix protein	SPON1	Q9HCB6	CSF	5	14	Stable isotope-LC-MS	PMID: 22232349
Superoxide dismutase 3, extracellular precursor	SOD3	P08294	CSF	5	14	Stable isotope-LC-MS	PMID: 22232349
Transferrin	TF	P02787	CSF	5	14	Stable isotope-LC-MS	PMID: 22232349
VGF nerve growth factor–inducible precursor	VGF	O15240	CSF	5	14	Stable isotope-LC-MS	PMID: 22232349
T-Tau	MAPT	P10636	CSF	13018	156 99	Meta-analysis	PMID: 27068280
P-Tau	MAPT	P10636	CSF	13018	156 99	Meta-analysis	PMID: 27068280
Neurofilament light polypeptide	NFL	P07196	CSF	13018	156 99	Meta-analysis	PMID: 27068280
Abeta 42	APP	P05067	CSF	13018	156 99	Meta-analysis	PMID: 27068280
Neuron-specific enolase	NSE	P09104	CSF	13018	156 99	Meta-analysis	PMID: 27068280
Visinin-like protein 1	VLP-1	P62760	CSF	13018	156 99	Meta-analysis	PMID: 27068280
Heart fatty acid binding protein	HFABP	P05413	CSF	13018	156 99	Meta-analysis	PMID: 27068280
Chitinase-3-like protein 1	YKL-40	P36222	CSF	13018	156 99	Meta-analysis	PMID: 27068280

CSF: Cerebrospinal Fluid
