# Supplementary material for: Drug Repositioning for Alzheimer’s Disease Based on Systematic ‘omics’ Data Mining
Source: PLoS One. 2016 Dec 22;11(12):e0168812. doi: 10.1371/journal.pone.0168812 (PMC5179106; doi:10.1371/journal.pone.0168812)
Supplement: S4 Table — (PDF) [file pone.0168812.s004.pdf]

S4 Table. AD associated metabolites retrieved from the HMDB database.

HMDB ID	Metabolite	Biofluid	Concentration	Age*	Sex	PUBMED ID
HMDB00001	1-Methylhistidine	Urine	15.7 +/- 4.04 umol/mmol creatinine	Adult	Both	17031479
HMDB00001	1-Methylhistidine	CSF	2.6 +/- 1.13 uM	Adult	Both	17031479
HMDB00001	1-Methylhistidine	Blood	10.66 +/- 2.68 uM	Elderly	Both	17031479
HMDB02712	1,5-Anhydrosorbitol	CSF	41.7 +/-13.7 uM	Adult	Both	8595727
HMDB01419	24-Hydroxycholesterol	CSF	0.0061 (0.0058-0.0064) uM	Elderly	Both	15061359
HMDB02103	27-Hydroxycholesterol	CSF	0.0038 +/- 0.0003 uM	Elderly	Both	15061359
HMDB00479	3-Methylhistidine	Urine	24.0 +/- 1.7 umol/mmol creatinine	Adult	Both	17031479
HMDB00479	3-Methylhistidine	CSF	2.48 +/- 1.58 uM	Adult	Not Specified	17031479
HMDB00479	3-Methylhistidine	Blood	5.16 +/- 1.27 uM	Elderly	Both	17031479
HMDB01336	3,4-Dihydroxybenzeneacetic acid	CSF	0.0023 (0.00051-0.0042) uM	Adult	Not Specified	10494443
HMDB06055	4-Hydroxy-L-proline	Urine	0.21 +/- 0.017 umol/mmol creatinine	Adult	Both	17031479
HMDB06055	4-Hydroxy-L-proline	CSF	0.28 +/- 0.055 uM	Adult	Both	17031479
HMDB06055	4-Hydroxy-L-proline	Blood	5.6 +/- 0.67 uM	Adult	Both	17031479
HMDB04362	4-Hydroxynonenal	CSF	0.40 +/- 0.030 uM	Adult	Not Specified	11959400
HMDB04362	4-Hydroxynonenal	CSF	0.36 +/- 0.03 uM	Adult	Both	11959400
HMDB00725	4-Hydroxyproline	Blood	5.66 +/- 0.67 uM	Elderly	Both	17031479
HMDB00450	5-Hydroxylysine	Blood	0.50 +/- 0.028 uM	Elderly	Both	17031479
HMDB02032	8-Hydroxyguanine	CSF	0.19 (0.15-0.23) uM	Adult	Not Specified	11255442
HMDB02044	8-Hydroxyguanosine	CSF	0.00050 +/- 0.00021 uM	Elderly	Both	12391605
HMDB02044	8-Hydroxyguanosine	Blood	0.00153 +/- 0.00060 uM	Adult	Both	12391605
HMDB00895	Acetylcholine	CSF	0.01 +/- 0.005 uM	Adult	Not Specified	15361288
HMDB01247	Aluminum	Blood	0.0823 +/- 0.0460 uM	Elderly	Both	16244393
HMDB00194	Anserine	Urine	8.0 +/- 5.0 umol/mmol creatinine	Adult	Both	17031479
HMDB00194	Anserine	Blood	0.457 +/- 0.214 uM	Elderly	Both	17031479
HMDB04142	Barium	Blood	0.0042 +/- 0.0019 uM	Elderly	Both	16244393
HMDB02387	Beryllium	Blood	0.0344 +/- 0.0189 uM	Elderly	Both	16244393
HMDB02196	Bismuth	Blood	0.000096 +/- 0.000048 uM	Elderly	Both	16244393
HMDB03638	Cadmium	Blood	0.00120 +/- 0.00053 uM	Elderly	Both	16244393
HMDB00464	Calcium	Blood	1714 +/- 132 uM	Elderly	Both	16244393

HMDB00033	Carnosine	Urine	18.8 +/- 10.31 umol/mmol creatinine	Adult	Both	17031479
HMDB00033	Carnosine	Blood	3.28 +/- 0.91 uM	Elderly	Both	17031479
HMDB00097	Choline	CSF	2.5 +/- 1.0 uM	Elderly	Both	15465626
HMDB00904	Citrulline	Urine	21.0 +/- 2.8 umol/mmol creatinine	Adult	Both	17031479
HMDB00904	Citrulline	CSF	27.0 +/- 2.6 uM	Adult	Both	17031479
HMDB00904	Citrulline	Blood	94.97 +/- 11.18 uM	Elderly	Both	17031479
HMDB00608	Cobalt	Blood	0.0019 +/- 0.0010 uM	Adult	Both	16244393
HMDB00657	Copper	CSF	1.39 +/- 1.02 uM	Elderly	Both	9720975
HMDB00657	Copper	Blood	15.4 +/- 3.9 uM	Elderly	Both	16244393
HMDB00122	D-Glucose	CSF	3700.0 (3200.0-4200.0) uM	Adult	Both	9693263
HMDB00609	DL-Dopa	Blood	14.0 +/- 2.5 uM	Adult	Both	17031479
HMDB00073	Dopamine	CSF	0.000078 (0.0000065-0.00015) uM	Adult	Both	10494443
HMDB00073	Dopamine	CSF	0.34 +/- 0.10 uM	Adult	Both	17031479
HMDB00073	Dopamine	Blood	18.0284 +/- 2.4552 uM	Elderly	Both	17031479
HMDB03869	Epsilon-(gamma-Glutamyl)-lysine	Blood	0.1629 +/- 0.0392 uM	Elderly	Both	17031479
HMDB00692	Fe2+	CSF	2.5 +/- 1.8 uM	Elderly	Not Specified	9720975
HMDB00692	Fe2+	CSF	1.8 +/- 1.8 uM	Elderly	Both	9720975
HMDB00692	Fe2+	Blood	16.3 +/- 7.7 uM	Elderly	Both	16244393
HMDB00121	Folic acid	CSF	0.0056 +/- 0.005 uM	Adult	Both	11959400
HMDB00121	Folic acid	CSF	0.008 +/- 0.0011 uM	Elderly	Both	11959400
HMDB00134	Fumaric acid	CSF	11.0 (2.00-20.0) uM	Elderly	Both	9693263
HMDB00107	Galactitol	CSF	1.65 +/- 0.22 uM	Adult	Both	8595727
HMDB00112	Gamma-Aminobutyric acid	Blood	1.84 +/- 0.215 uM	Elderly	Both	17031479
HMDB00086	Glycerophosphocholine	CSF	6.9 +/- 3.2 uM	Elderly	Both	15465626
HMDB00123	Glycine	Urine	0.010 +/- 0.001 umol/mmol creatinine	Adult	Both	17031479
HMDB00123	Glycine	CSF	0.28 +/- 0.12 uM	Adult	Both	17031479
HMDB00123	Glycine	Blood	0.153 +/- 0.012 uM	Elderly	Both	17031479
HMDB00721	Glycylproline	Urine	0.0087 +/- 0.0016 umol/mmol creatinine	Elderly	Both	17031479
HMDB00721	Glycylproline	CSF	0.086 +/- 0.016 uM	Elderly	Both	17031479
HMDB00721	Glycylproline	Blood	0.23 +/- 0.04 uM	Elderly	Both	17031479
HMDB00742	Homocysteine	CSF	14.2 +/- 1.7 uM	Elderly	Not Specified	11959400
HMDB00742	Homocysteine	CSF	0.12 +/- 0.062 uM	Elderly	Not Specified	16227558
HMDB06284	L-2,4-diaminobutyric acid	Urine	36.0 +/- 5.0 umol/mmol creatinine	Adult	Both	17031479
HMDB06284	L-2,4-diaminobutyric	Blood	116.35 +/- 10.0 uM	Adult	Both	17031479

	acid					
HMDB00452	L-Alpha-aminobutyric acid	Blood	19.79 +/- 2.17 uM	Adult	Both	17031479
HMDB01851	L-Arabitol	CSF	22.4 +/- 4.6 uM	Adult	Both	8595727
HMDB00517	L-Arginine	Urine	2.0 +/- 0.21 umol/mmol creatinine	Adult	Both	17031479
HMDB00517	L-Arginine	CSF	6.0 +/- 1.4 uM	Adult	Both	17031479
HMDB00517	L-Arginine	Blood	91.34 +/- 13.1 uM	Elderly	Both	17031479
HMDB00168	L-Asparagine	CSF	1.77 +/- 0.16 umol/mmol creatinine	Adult	Both	17031479
HMDB00191	L-Aspartic acid	Blood	12.67 +/- 2.86 uM	Elderly	Both	17031479
HMDB00099	L-Cystathionine	Urine	1.68 +/- 0.5 umol/mmol creatinine	Adult	Both	17031479
HMDB00099	L-Cystathionine	Blood	1.03 +/- 0.22 uM	Elderly	Both	17031479
HMDB00192	L-Cystine	Urine	72.03 +/- 12.0 umol/mmol creatinine	Adult	Both	17031479
HMDB00192	L-Cystine	CSF	7.8 +/- 2.5 umol/mmol creatinine	Adult	Both	17031479
HMDB00192	L-Cystine	Blood	209.0 +/- 46.0 uM	Elderly	Both	17031479
HMDB00181	L-Dopa	Blood	14.0 +/- 2.53 uM	Elderly	Both	17031479
HMDB00148	L-Glutamic acid	Blood	33.18 +/- 11.26 uM	Elderly	Both	17031479
HMDB00641	L-Glutamine	Urine	1.17 +/- 0.49 umol/mmol creatinine	Adult	Both	17031479
HMDB00641	L-Glutamine	CSF	254.0 (152.0-356.0) uM	Adult	Both	9693263
HMDB00641	L-Glutamine	CSF	1146.6 +/- 10.6 uM	Adult	Both	17031479
HMDB00641	L-Glutamine	Blood	228.34 +/- 12.00 uM	Elderly	Both	17031479
HMDB00177	L-Histidine	Urine	30.5 +/- 4.3 umol/mmol creatinine	Adult	Both	17031479
HMDB00177	L-Histidine	CSF	14.4 +/- 6.32 uM	Adult	Both	17031479
HMDB00177	L-Histidine	Blood	116.68 +/- 18.15 uM	Elderly	Both	17031479
HMDB00172	L-Isoleucine	Urine	0.13 +/- 0.04 umol/mmol creatinine	Adult	Both	17031479
HMDB00172	L-Isoleucine	CSF	2.4 +/- 0.65 uM	Adult	Both	17031479
HMDB00172	L-Isoleucine	Blood	1495.2 +/- 373.0 uM	Elderly	Both	17031479
HMDB00687	L-Leucine	Urine	0.91 +/- 0.11 umol/mmol creatinine	Adult	Both	17031479
HMDB00687	L-Leucine	CSF	11.5 +/- 1.5 uM	Adult	Both	17031479
HMDB00182	L-Lysine	Urine	5.5 +/- 1.74 umol/mmol creatinine	Adult	Both	17031479
HMDB00182	L-Lysine	CSF	44.6 +/- 10.8 uM	Adult	Both	17031479
HMDB00182	L-Lysine	Blood	216.7 +/- 54.5 uM	Elderly	Both	17031479
HMDB00159	L-Phenylalanine	Urine	1.37 +/- 0.18 umol/mmol creatinine	Adult	Both	17031479
HMDB00159	L-Phenylalanine	CSF	6.0 +/- 0.8 uM	Adult	Both	17031479
HMDB00159	L-Phenylalanine	Blood	71.77 +/- 9.21 uM	Elderly	Both	17031479
HMDB00162	L-Proline	CSF	0.68 +/- 0.35 uM	Adult	Both	17031479
HMDB00187	L-Serine	Blood	0.21 +/- 0.024 uM	Elderly	Both	17031479

HMDB00167	L-Threonine	Urine	0.03 +/- 0.003 umol/mmol creatinine	Adult	Both	17031479
HMDB00929	L-Tryptophan	Urine	1.19 +/- 0.23 umol/mmol creatinine	Adult	Both	17031479
HMDB00929	L-Tryptophan	CSF	1.3 +/- 0.32 uM	Adult	Both	17031479
HMDB00929	L-Tryptophan	Blood	33.06 +/- 8.99 uM	Adult	Both	17031479
HMDB00158	L-Tyrosine	Urine	10.5 +/- 1.5 umol/mmol creatinine	Adult	Both	17031479
HMDB00158	L-Tyrosine	CSF	27.18 +/- 4.8 uM	Adult	Both	17031479
HMDB00158	L-Tyrosine	Blood	143.77 +/- 15.49 uM	Elderly	Both	17031479
HMDB00883	L-Valine	Urine	0.73 +/- 0.12 umol/mmol creatinine	Adult	Both	17031479
HMDB00883	L-Valine	CSF	4.13 +/- 1.22 uM	Adult	Both	17031479
HMDB00883	L-Valine	Blood	58.26 +/- 14.74 uM	Elderly	Both	17031479
HMDB04628	Lead	Blood	0.0021 +/- 0.0013 uM	Elderly	Both	16244393
HMDB05949	Lithium	Blood	0.16 +/- 0.074 uM	Elderly	Both	16244393
HMDB00547	Magnesium	Blood	763 +/- 100 uM	Adult	Both	16244393
HMDB01333	Manganese	CSF	14.6 +/- 13.7 uM	Elderly	Not Specified	9720975
HMDB01333	Manganese	CSF	0.0146 +/- 0.0146 uM	Elderly	Both	9720975
HMDB01333	Manganese	Blood	0.011 +/- 0 .004 uM	Elderly	Both	16244393
HMDB00765	Mannitol	CSF	5.54 +/- 0.82 uM	Not Specified	Not Specified	8595727
HMDB00765	Mannitol	CSF	5.6 +/-1.1 uM	Adult	Both	8595727
HMDB03625	Mercury	Blood	0.0088 +/- 0.0039 uM	Elderly	Both	16244393
HMDB00202	Methylmalonic acid	CSF	0.30 +/- 0.12 uM	Elderly	Not Specified	16227558
HMDB00202	Methylmalonic acid	Blood	0.480 +/- 0.062 uM	Adult	Both	8356878
HMDB01302	Molybdenum	Blood	0.012 +/- 0.0059 uM	Elderly	Both	16244393
HMDB00211	Myoinositol	CSF	158.8 +/- 27.2 uM	Not Specified	Not Specified	8595727
HMDB00211	Myoinositol	CSF	158.8 +/- 27.2 uM	Adult	Both	8595727
HMDB02457	Nickel	Blood	0.01 +/- 0.0062 uM	Adult	Both	16244393
HMDB00214	Ornithine	Urine	12.34 +/- 5.07 umol/mmol creatinine	Adult	Both	17031479
HMDB00214	Ornithine	Blood	68.35 +/- 22.43 uM	Adult	Both	17031479
HMDB03933	Pentosidine	CSF	12.5 (12.0-13.0) uM	Adult	Not Specified	12498967
HMDB01565	Phosphorylcholine	CSF	2.16 +/- 0.84 uM	Elderly	Not Specified	15465626
HMDB06695	Prolylhydroxyproline	CSF	0.086 +/- 0.016 uM	Adult	Both	17031479
HMDB00508	Ribitol	CSF	3.8 +/- 0.68 uM	Elderly	Both	8595727
HMDB00508	Ribitol	Blood	0.55 +/- 0.08 uM	Elderly	Both	8595727
HMDB02175	Silicon	Blood	7.97 +/- 3.79 uM	Elderly	Both	16244393

HMDB00247	Sorbitol	CSF	22.89 +/- 3.34 uM	Not Specified	Not Specified	8595727
HMDB00247	Sorbitol	CSF	23.3 +/- 3.3 uM	Adult	Both	8595727
HMDB03642	Strontium	Blood	0.44 +/- 0.16 uM	Elderly	Both	16244393
HMDB00254	Succinic acid	CSF	55.0 (19.0-91.0) uM	Adult	Both	9693263
HMDB02666	Thiamine monophosphate	CSF	0.0035 +/- 0.0038 uM	Adult	Both	12111441
HMDB01372	Thiamine pyrophosphate	CSF	0.0025 +/- 0.0017 uM	Adult	Both	12111441
HMDB01960	Tin	Blood	0.011 +/- 0.0056 uM	Adult	Both	16244393
HMDB01966	Titanium	Blood	0.00083 +/- 0.00042 uM	Elderly	Both	16244393
HMDB01989	Tungsten	Blood	0.00016 +/- 0.00011 uM	Elderly	Both	16244393
HMDB01989	Tungsten	Blood	0.00033 +/- 0.00016 uM	Elderly	Both	16244393
HMDB02503	Vanadium	Blood	0.00098 +/- 0.00059 uM	Elderly	Both	16244393
HMDB01303	Zinc ion	CSF	1.83 +/- 1.38 uM	Elderly	Not Specified	9720975
HMDB01303	Zinc ion	CSF	1.6 +/- 1.6 uM	Adult	Both	9720975
HMDB01303	Zinc ion	Blood	10.7 +/- 1.8 uM	Elderly	Both	16244393

*Age: Adult >18 years old; Elderly >65 years old
CSF: Cerebrospinal Fluid
