# Supplementary material for: Drug Repositioning for Alzheimer’s Disease Based on Systematic ‘omics’ Data Mining
Source: PLoS One. 2016 Dec 22;11(12):e0168812. doi: 10.1371/journal.pone.0168812 (PMC5179106; doi:10.1371/journal.pone.0168812)
Supplement: S5 Table — (PDF) [file pone.0168812.s005.pdf]

S5 Table. AD related proteins from two platforms.

Gene name	GWAS	Proteomics	Epigenetics	Metabolomics	Functional studies
ABCA7	yes		yes		yes[PMID: 25807283]
APOE	yes	yes			yes[PMID: 24559670]
BIN1	yes		yes		yes[PMID: 23399914]
PICALM	yes		yes		yes[PMID: 26005850]
CELF1	yes		yes		
INPP5D	yes		yes		
SPON1	yes	yes			
SOD3		yes		yes	
