# Supplementary material for: Drug Repositioning for Alzheimer’s Disease Based on Systematic ‘omics’ Data Mining
Source: PLoS One. 2016 Dec 22;11(12):e0168812. doi: 10.1371/journal.pone.0168812 (PMC5179106; doi:10.1371/journal.pone.0168812)
Supplement: S6 Table — (PDF) [file pone.0168812.s006.pdf]

S6 Table. Potential anti-AD drug targets with existing approved or clinical trial drugs.

Uniprot ID	Database	Target name	Target score	Target source	Drug name	Previous Indication	Stage	MOA	AD pathogenesis
P20138	TTD	Myeloid cell surface antigen CD33	0.715	GWAS	Gemtuzumab ozogamicin	Acute myelogenous leukemia	Approved	Antibody	GOF lead to the impairment of microglia-mediated clearance of Aβ[PMID: 23708142]
P20138	TTD	Myeloid cell surface antigen CD33	0.715	GWAS	Vadastuximab talirine	Acute myelogenous leukemia	Phase I/II	Antibody	GOF lead to the impairment of microglia-mediated clearance of Aβ[PMID: 23708142]
P20138	TTD	Myeloid cell surface antigen CD33	0.715	GWAS	Lintuzumab	Leukemia	Phase II discontinued	Antibody	GOF lead to the impairment of microglia-mediated clearance of Aβ[PMID: 23708142]
P20138	TTD	Myeloid cell surface antigen CD33	0.715	GWAS	BI-836858	Acute myelogenous leukemia	Phase I	Antibody	GOF lead to the impairment of microglia-mediated clearance of Aβ[PMID: 23708142]
P20138	TTD	Myeloid cell surface antigen CD33	0.715	GWAS	HuM195/rGel	Leukemia	Phase I	Antibody	GOF lead to the impairment of microglia-mediated clearance of Aβ[PMID: 23708142]
P20138	TTD	Myeloid cell surface antigen CD33	0.715	GWAS	HuM-195-Ac-225	Acute myelogenous leukemia	Phase I/II	Antibody	GOF lead to the impairment of microglia-mediated clearance of Aβ[PMID: 23708142]
P04233	TTD	Macrophage migration inhibitory factor	0.4	Proteomics	Anti-MIF antibody	Solid tumor	Phase I	Antibody	LOF reduces Aβ-induced toxicity[PMID: 20200619]

P22303	DrugBank	Acetylcholinesterase*	0.372	Metabolomics	Galantamine[#]	Alzheimer's disease	Approved	Inhibitor	LOF improves AD[PMID: 16437532]
P22303	DrugBank	Acetylcholinesterase *	0.372	Metabolomics	Rivastigmine[#]	Alzheimer's Disease	Approved	Inhibitor	LOF improves AD[PMID: 16437532]
P22303	DrugBank	Acetylcholinesterase *	0.372	Metabolomics	Donepezil[#]	Alzheimer's Disease	Approved	Modulator	LOF improves AD[PMID: 16437532]
P22303	DrugBank	Acetylcholinesterase *	0.372	Metabolomics	Gallamine Triethiodide	Adjunct to anesthesia	Approved	Inhibitor	LOF improves AD[PMID: 16437532]
P22303	DrugBank	Acetylcholinesterase *	P22303	DrugBank	Pyridostigmine	Myasthenia gravis	Approved	Inhibitor	LOF improves AD[PMID: 16437532]
P22303	DrugBank	Acetylcholinesterase *	P22303	DrugBank	Demecarium	Glaucoma	Approved	Inhibitor	LOF improves AD[PMID: 16437532]
P22303	DrugBank	Acetylcholinesterase *	P22303	DrugBank	Physostigmine	Glaucoma	Approved	Inhibitor	LOF improves AD[PMID: 16437532]
P22303	DrugBank	Acetylcholinesterase *	P22303	DrugBank	Endrophonium	Myasthenia gravis	Approved	Inhibitor	LOF improves AD[PMID: 16437532]
P22303	DrugBank	Acetylcholinesterase *	P22303	DrugBank	Ambenonium	Muscle weakness	Approved	Inhibitor	LOF improves AD[PMID: 16437532]
P22303	DrugBank	Acetylcholinesterase *	P22303	DrugBank	Isoflurophate	Glaucoma	Approved, withdrawn	Inhibitor	LOF improves AD[PMID: 16437532]
Q96KS0	TTD	Hypoxia-inducible factor-prolyl hydroxylase	0.319	Metabolomics	FG-2216	Anemia, Kidney Disease	Phase II completed	Inhibitor	LOF protects neuron[PMID: 16227210]
Q96KS0	TTD	Hypoxia-inducible factor-prolyl hydroxylase	0.319	Metabolomics	FG-4592	Anemia, Kidney Disease	Phase II	Inhibitor	LOF protects neuron[PMID: 16227210]
Q96KS0	TTD	Hypoxia-inducible factor-prolyl hydroxylase	0.319	Metabolomics	GSK1278863	Anaemia	Phase I completed	Inhibitor	LOF protects neuron[PMID: 16227210]
Q96KS0	TTD	Hypoxia-inducible factor-prolyl hydroxylase	0.319	Metabolomics	BAY 85-3934	Anemia	Phase I	Inhibitor	LOF protects neuron[PMID: 16227210]
O43497	TTD	Voltage-dependent T-type calcium channel alpha-1G subunit	0.291	GWAS	Ethosuximide	Epilepsy	Approved	Blocker	LOF induces Aβ[PMID: 24268883]

O43497	TTD	Voltage-dependent T-type calcium channel alpha-1G subunit	0.291	GWAS	Methsuximide	Epileptic seizures	Approved	Blocker	LOF induces Aβ[PMID: 24268883]
O43497	TTD	Voltage-dependent T-type calcium channel alpha-1G subunit	0.291	GWAS	Paramethadione	Paramethadione syndrome	Approved	Blocker	LOF induces Aβ[PMID: 24268883]
O43497	TTD	Voltage-dependent T-type calcium channel alpha-1G subunit	0.291	GWAS	Trimethadione	Epileptic conditions	Approved	Blocker	LOF induces Aβ[PMID: 24268883]
O43497	TTD	Voltage-dependent T-type calcium channel alpha-1G subunit	0.291	GWAS	Verapamil	Hypertension and angina	Approved	Blocker	LOF induces Aβ[PMID: 24268883]
O43497	TTD	Voltage-dependent T-type calcium channel alpha-1G subunit	0.291	GWAS	Mibefradil	Hypertension	Approved, withdrawn	Blocker	LOF induces Aβ[PMID: 24268883]
P00747	TTD/Drug Bank	Plasminogen	0.192	Proteomics	Alteplase	Pulmonary embolism	Approved	Activator	GOF reduces Aβ[PMID: 24126163]
P00747	TTD/Drug Bank	Plasminogen	0.192	Proteomics	Anistreplase	Acute Coronary thrombosis	Approved	Activator	GOF reduces Aβ[PMID: 24126163]
P00747	TTD/Drug Bank	Plasminogen	0.192	Proteomics	Reteplase	Heart attack	Approved	Activator	GOF reduces Aβ[PMID: 24126163]
P00747	TTD/Drug Bank	Plasminogen	0.192	Proteomics	Streptokinase	Pulmonary embolism	Approved	Activator	GOF reduces Aβ[PMID: 24126163]
P00747	TTD/Drug Bank	Plasminogen	0.192	Proteomics	Tenecteplase	Myocardial infarction	Approved	Activator	GOF reduces Aβ[PMID: 24126163]
P00747	TTD	Plasminogen	0.192	Proteomics	Tranexamic Acid	Excessive bleeding	Approved	Activator	GOF reduces Aβ[PMID: 24126163]
P00747	TTD/Drug Bank	Plasminogen	0.192	Proteomics	Urokinase	Deep venous thrombosis; Pulmonary embolism; Myocardial infarction; Occluded intravenous or dialysis cannulas	Approved	Activator	GOF reduces Aβ[PMID: 24126163]

P21728	TTD/Drug Bank	Dopamine D1 receptor	0.171	Metabolomi cs	Fenoldopam	Hypertension	Approved	Agonist	GOF protects neuron[PMID: 21387370]
P21728	TTD/Drug Bank	Dopamine D1 receptor	0.171	Metabolomi cs	Pergolide	Parkinson's disease	Approved	Agonist	GOF protects neuron[PMID: 21387370]
P21728	DrugBank	Dopamine D1 receptor	0.171	Metabolomi cs	Cabergoline	Hyperprolactinemic disorders	Approved	Agonist	GOF protects neuron[PMID: 21387370]
P21728	DrugBank	Dopamine D1 receptor	0.171	Metabolomi cs	Ropinirole	Parkinson's disease	Approved	Agonist	GOF protects neuron[PMID: 21387370]
P21728	DrugBank	Dopamine D1 receptor	0.171	Metabolomi cs	Phenylpropan olamine	Obesity	Approved, withdrawn	Partial agonist	GOF protects neuron[PMID: 21387370]
P21728	DrugBank	Dopamine D1 receptor	0.171	Metabolomi cs	Apomorphine	Parkinson's Disease	Approved	Agonist	GOF protects neuron[PMID: 21387370]
P21728	DrugBank	Dopamine D1 receptor	0.171	Metabolomi cs	Minaprine	Depression	Approved	Agonist	GOF protects neuron[PMID: 21387370]
P21728	DrugBank	Dopamine D1 receptor	0.171	Metabolomi cs	Dopamine	Parkinson's Disease	Approved	Agonist	GOF protects neuron[PMID: 21387370]
P21728	DrugBank	Dopamine D1 receptor	0.171	Metabolomi cs	Bromocriptine	Parkinson's disease	Approved	Agonist	GOF protects neuron[PMID: 21387370]
P21728	DrugBank	Dopamine D1 receptor	0.171	Metabolomi cs	Levodopa	Parkinson's disease	Approved	Agonist	GOF protects neuron[PMID: 21387370]
P21728	DrugBank	Dopamine D1 receptor	0.171	Metabolomi cs	Rotigotine	Motor symptoms and Parkinson's disease	Approved	Agonist	GOF protects neuron[PMID: 21387370]
P21728	DrugBank	Dopamine D1 receptor	0.171	Metabolomi cs	Cabergoline	Hyperprolactinemic disorders	Approved	Agonist	GOF protects neuron[PMID: 21387370]
P21728	DrugBank	Dopamine D1 receptor	0.171	Metabolomi cs	Ergotamine	Headache	Approved	Agonist	GOF protects neuron[PMID: 21387370]
P00325	DrugBank	Alcohol dehydrogenase 1B	0.159	Metabolomi cs	Fomepizole	Athylene glycol or methanol poisoning	Approved	Inhibitor	GOF contributes to pathogenesis of amyloid[PMID: 15910550]
P01009	TTD	Alpha-1-antitrypsin	0.155	Proteomics	lpha1-proteinase inhibitor	Emphysema	Approved	Inhibitor	GOF induces AD pathogenesis[PMID: 1623174]
P01009	TTD	Alpha-1-antitrypsin	0.155	Proteomics	Zemaira	Emphysema	Approved	Inhibitor	GOF induces AD pathogenesis[PMID: 1623174]

P01009	TTD	Alpha-1-antitrypsin	0.155	Proteomics	Alphagen	Chronic obstructive pulmonary disease, cystic fibrosis	Phase I	Inhibitor	GOF induces AD pathogenesis[PMID: 1623174]
P35228	TTD	Nitric oxide synthase, inducible	0.146	Metabolomics	Hydrocortisone	Inflammatory diseases	Approved	Inhibitor	LOF protects AD mice from increased Aβ levels[PMID: 16260491]
P35228	DrugBank	Nitric oxide synthase, inducible	0.146	Metabolomics	Miconazole	Fungal infections	Approved	Inhibitor	LOF protects AD mice from increased Aβ levels[PMID: 16260491]
P35228	DrugBank	Nitric oxide synthase, inducible	0.146	Metabolomics	Dexamethasone	Rheumatoid arthritis	Approved	Negative modulator	LOF protects AD mice from increased Aβ levels[PMID: 16260491]
P05164	TTD	Myeloperoxidase	0.144	Metabolomics	AZD3241	Parkinson's disease	Phase I	Inhibitor	GOF induces lipid peroxidation in AD[PMID: 19059911]
P05164	TTD	Myeloperoxidase	0.144	Metabolomics	AZD5904	Multiple sclerosis	Discontinued in Phase I	Inhibitor	GOF induces lipid peroxidation in AD[PMID: 19059911]
P15692	TTD	Vascular endothelial growth factor A	0.144	Proteomics	Ziv-aflibercept	Metastatic colorectal cancer	Approved	Inhibitor	LOF induces AD [PMID: 9795165]
P15692	TTD	Vascular endothelial growth factor A	0.144	Proteomics	RG7221	Oncology	Phase I	Antibody	LOF induces AD [PMID: 9795165]
P15121	TTD	Aldose reductase	0.139	Metabolomics	Epalrestat	Diabetic neuropathy	Approved	Inhibitor	GOF induces AD[PMID: 11444797]
P15121	TTD/Drug Bank	Aldose reductase	0.139	Metabolomics	Sulindac	Rheumatoid arthritis	Approved	Inhibitor	GOF induces AD[PMID: 11444797]
P15121	TTD	Aldose reductase	0.139	Metabolomics	Fidarestat	Diabetes	Phase III	Inhibitor	GOF induces AD[PMID: 11444797]
P15121	TTD	Aldose reductase	0.139	Metabolomics	AS-3201	Diabetic neuropathy	(US/EU) PII/III	Inhibitor	GOF induces AD[PMID: 11444797]
P15121	TTD	Aldose reductase	0.139	Metabolomics	Sorbinil	Diabetes	Discontinued after Phase III	Inhibitor	GOF induces AD[PMID: 11444797]
P15121	TTD	Aldose reductase	0.139	Metabolomics	Zenarestat	Diabetic Neuropathy	Discontinued	Inhibitor	GOF induces AD[PMID: 11444797]

O76074	TTD	CGMP-specific 3',5'-cyclic phosphodiesterase	0.138	Metabolomics	Papaverine	Spasm	Approved	Inhibitor	LOF improves cognitive deficiency[PMID: 23173065]
O76074	TTD	CGMP-specific 3',5'-cyclic phosphodiesterase	0.138	Metabolomics	Sildenafil	Erectile dysfunction	Approved	Inhibitor	LOF improves cognitive deficiency[PMID: 23173065]
O76074	TTD	CGMP-specific 3',5'-cyclic phosphodiesterase	0.138	Metabolomics	Tadalafil	Erectile dysfunction	Approved	Inhibitor	LOF improves cognitive deficiency[PMID: 23173065]
O76074	TTD	CGMP-specific 3',5'-cyclic phosphodiesterase	0.138	Metabolomics	Udenafil	Erectile dysfunction	Approved	Inhibitor	LOF improves cognitive deficiency[PMID: 23173065]
O76074	TTD	CGMP-specific 3',5'-cyclic phosphodiesterase	0.138	Metabolomics	Vardenafil	Erectile dysfunction	Approved	Inhibitor	LOF improves cognitive deficiency[PMID: 23173065]
O76074	TTD	CGMP-specific 3',5'-cyclic phosphodiesterase	0.138	Metabolomics	PF-00489791	Diabetic nephropathy	Phase II	Inhibitor	LOF improves cognitive deficiency[PMID: 23173065]
O14939	DrugBank	Phospholipase D2	0.137	Metabolomics	Lithium	Bipolar Disorder	Approved	Inhibitor	LOF ameliorates AD symptom[PMID: 21147981]
P21917	DrugBank	Dopamine D4 receptor	0.133	Metabolomics	Pramipexole	Parkinson's disease	Approved	Agonist	LOF induces cognitive dysfunction[PMID: 22815864]
P21917	DrugBank	Dopamine D4 receptor	0.133	Metabolomics	Lisuride	Parkinson's disease	Approved	Agonist	LOF induces cognitive dysfunction[PMID: 22815864]
P21917	DrugBank	Dopamine D4 receptor	0.133	Metabolomics	Aripiprazole	Schizophrenia	Approved	Partial agonist	LOF induces cognitive dysfunction[PMID: 22815864]
P21964	TTD/DrugBank	Catechol-O-methyl-transferase	0.13	Metabolomics	Entacapone	Parkinson's disease	Approved	Inhibitor	LOF blocks Amyoid fibril[PMID: 22483294]
P21964	TTD/DrugBank	Catechol-O-methyl-transferase	0.13	Metabolomics	Tolcapone	Parkinson's disease	Approved	Inhibitor	LOF blocks Amyoid fibril[PMID: 22483294]
P21964	DrugBank	Catechol-O-methyl-transferase	0.13	Metabolomics	Testosterone Propionate	muscle mass building	Approved	Inhibitor	LOF blocks Amyoid fibril[PMID: 22483294]
P10635	TTD	Cytochrome P450 2D6	0.128	Metabolomics	Glutethimide	Insomnia	Approved	Inducer	LOF induces AD[Tanata et al., 2011]

P05067	TTD	Amyloid precursor protein*	1	Proteomics	Solanezumab#	Slow the progression of Alzheimer's disease	Phase III	Antibody	Mutation causes AD[PMID: 1671712]
P05067	TTD	Amyloid precursor protein*	1	Proteomics	933776#	Geographic retinal atrophy, Alzheimer's disease	Phase II	Antibody	Mutation causes AD[PMID: 1671712]
P05067	TTD	Amyloid precursor protein*	1	Proteomics	Anti-Abeta#	Alzheimer's disease	Phase II	Antibody	Mutation causes AD[PMID: 1671712]
P05067	TTD	Amyloid precursor protein*	1	Proteomics	BAN2401#	Alzheimer's disease	(US/EU) PII	Antibody	Mutation causes AD[PMID: 1671712]
P05067	TTD	Amyloid precursor protein*	1	Proteomics	CAD106#	Alzheimer's disease	Phase II	Antibody	Mutation causes AD[PMID: 1671712]
P05067	TTD	Amyloid precursor protein*	1	Proteomics	Crenezumab#	Alzheimer's Disease	Phase II	Antibody	Mutation causes AD[PMID: 1671712]
P05067	TTD	Amyloid precursor protein*	1	Proteomics	ELND005#	Mild to moderate Alzheimer's disease (Fast Track)	Phase II	Neutralize	Mutation causes AD[PMID: 1671712]
P05067	TTD	Amyloid precursor protein*	1	Proteomics	RG1450#	Alzheimer's disease	Phase II	Antibody	Mutation causes AD[PMID: 1671712]
P05067	TTD	Amyloid precursor protein*	1	Proteomics	RG7412#	Alzheimer's disease	Phase II	Antibody	Mutation causes AD[PMID: 1671712]
P05067	TTD	Amyloid precursor protein*	1	Proteomics	GSK933776A#	Alzheimer's disease	Phase I	Antibody	Mutation causes AD[PMID: 1671712]
P05067	TTD	Amyloid precursor protein*	1	Proteomics	AAB-003/PF-05236812#	Alzheimer's disease	Phase I	Antibody	Mutation causes AD[PMID: 1671712]
P05067	TTD	Amyloid precursor protein*	1	Proteomics	BAN2401#	Mild to moderate Alzheimer's disease	Phase I	Antibody	Mutation causes AD[PMID: 1671712]
P05067	TTD	Amyloid precursor protein*	1	Proteomics	BIIB037#	Alzheimer's Disease	Phase I	Antibody	Mutation causes AD[PMID: 1671712]
P05067	TTD	Amyloid precursor protein*	1	Proteomics	R1450#	Alzheimer's disease	Phase I	Antibody	Mutation causes AD[PMID: 1671712]

*known anti-AD target; #approved or clinical trial drugs for AD; MOA: mode of action; GOF: gain of function; LOF: loss of function
