# Supplementary material for: Drug Repositioning for Alzheimer’s Disease Based on Systematic ‘omics’ Data Mining
Source: PLoS One. 2016 Dec 22;11(12):e0168812. doi: 10.1371/journal.pone.0168812 (PMC5179106; doi:10.1371/journal.pone.0168812)
Supplement: S7 Table — Target score was calculated using weighted values of 0.33, 0.34, 0.33 for fold change/odds ratio, number of citations for the paper reporting the pathogenic mechanism, and number of reports on AD and target gene, respectively. Toppgene and Toppnet scores were estimated to validate our ranking algorithm. (PDF) [file pone.0168812.s007.pdf]

S7 Table. Ranking of the potential AD drug targets. Target score was calculated using weighted values of 0.33, 0.34, 0.33 for fold change/odds ratio, number of citations for the paper reporting the pathogenic mechanism, and number of reports on AD and target gene, respectively. Toppgene and Toppnet scores were estimated to validate our ranking algorithm.

Uniprot ID	Target Name	Fold change or odds ratio	Number of citations for the paper reporting the pathogenic mechanism	Number of reports on AD and target gene	Target score	Rank	Toppgene score	Toppnet score
P20138	Myeloid cell surface antigen CD33	5.55	93	39	0.782	1	**0.67**	**6.70E-05**
P04233	Macrophage migration inhibitory factor	3.13	28	12	0.438	2	**0.71**	**1.40E-05**
P22303	Acetylcholinesterase*	-3.45, -3.33	1313	4259	0.384	3	**0.83**	**2.80E-05**
Q96KS0	Hypoxia-inducible factor-prolyl hydroxylase	-2.2, -5.9, 2.9	238	1	0.345	4	0.55	**3.40E-05**
O43497	Voltage-dependent T-type calcium channel alpha-1G subunit	0.28	8	1	0.319	5	**0.63**	2.40E-06
P00747	Plasminogen	1.5	7	102	0.212	6	**0.87**	**1.20E-04**
P21728	Dopamine D1 receptor	1.06	330	259	0.183	7	**0.69**	**1.90E-05**
P00325	Alcohol dehydrogenase 1B	-3.3, 3.66	102	55	0.170	8	0.45	8.80E-06
P01009	Alpha-1-antitrypsin	1.11	105	56	0.165	9	**0.86**	**1.50E-04**
P15692	Vascular endothelial growth factor A	-1.05	174	104	0.164	10	**0.85**	**4.10E-05**
P05164	Myeloperoxidase	-2, -1.8	43	311	0.161	11	**0.84**	4.60E-06
O14939	Phospholipase D2	-3.3, 3.66	80	3	0.159	12	**0.67**	**7.60E-05**
P21917	Dopamine D4 receptor	1.06	15	259	0.156	13	**0.75**	**1.00E-05**
P35228	Nitric oxide synthase, inducible	-1.05, -1.19	124	139	0.155	14	**0.67**	**1.45E-04**
P15121	Aldose reductase	1, -1.28, 1.19	78	12	0.149	15	0.45	8.80E-06
O76074	CGMP-specific 3',5'-cyclic phosphodiesterase	-1.64, -1.05	46	3	0.149	16	**0.63**	**4.10E-05**
P10635	Cytochrome P450 2D6	1.06	0	58	0.149	17	**0.62**	**2.40E-05**
P21964	Catechol-O-methyl-transferase	1.15, 1.06, -1.3	9	39	0.143	18	**0.65**	**2.30E-05**
P05067	Amyloid precursor protein[#]	-2.37	4092	11294	1.000	NA	NA	NA
P02649	Apolipoprotein E e4*	3.7	3691[@]	8574	0.887	NA	NA	NA

Q9NZC2	TREM2*	5.05	73	104	0.459	NA	NA	NA

[#] internal control; [*]positive control; [@] reference : PMID8446617
Medium/high Toppgene scores (>0.6) and Toppnet scores (>1.0E-05) were shown in bold.
